# The Intestinal Barrier: A Multilayered Gatekeeper Against Systemic Disease

**DOI:** 10.1155/ijm/2828137

**Published:** 2026-06-09

**Authors:** Jun Wakabayashi, Katsunori Kimura, Takeshi Kawauchi

**Affiliations:** ^1^ Department of Adaptive and Maladaptive Responses in Health and Disease, Graduate School of Medicine, Kyoto University, Kyoto, Japan, kyoto-u.ac.jp; ^2^ Wellness Science Labs, Meiji Holdings Co., Ltd., Hachioji, Tokyo, Japan; ^3^ Department of Physiology, Keio University School of Medicine, Shinjuku-ku, Tokyo, Japan, keio.ac.jp

**Keywords:** chemical barrier, gut–brain axis, gut–kidney axis, gut–liver axis, gut–muscle axis, immunological barrier, intestinal barrier, leaky gut, microbiological barrier, physical barrier

## Abstract

The intestine performs two distinct yet seemingly contradictory functions: the digestion and absorption of nutrients, and the defense against harmful substances and pathogens. The intestinal barrier is a multilayered system maintained by four key components: the physical, chemical, microbiological, and immunological barriers. Each layer plays a unique role in preventing the translocation of harmful entities from the intestinal lumen into the body. Disruption of this integrated barrier increases intestinal permeability, a condition commonly referred to as “leaky gut,” in which harmful substances and pathogens can enter the systemic circulation. We also highlight that barrier dysfunction differs between the small and large intestines, reflecting regional differences in epithelial structure, mucus architecture, and microbial load. Growing evidence indicates that such barrier disruption triggers cascades of inflammatory responses in various organs, including the liver, brain, muscles, and kidneys, leading to impaired physiological functions. This review delineates the four layers of the intestinal barrier and explores the systemic consequences of its disruption. In addition, it summarizes current approaches for assessing intestinal barrier function and discusses the strengths and limitations of widely used experimental models. The review also outlines current therapeutic strategies for barrier restoration, including dietary interventions, microbiota‐directed approaches, postbiotics, bile acid modulators, anti‐inflammatory and immunomodulatory therapies, and lifestyle modification. A deeper understanding of this multilayered gatekeeper is essential for developing novel preventive and therapeutic strategies aimed at restoring barrier integrity and strengthening the defense against a wide range of systemic diseases.

## 1. Introduction

Although the primary functions of the intestinal tract are digesting food and absorbing nutrients and water, the intestinal tract possesses a crucial defense mechanism that prevents harmful substances and pathogens from entering the body [1]. This system, known as an intestinal barrier, plays a vital role in host defense. The intestinal barrier is composed of four main components: physical, chemical, microbiological, and immunological barriers, each of which contributes to its integrity through distinct mechanisms [2–4]. However, the intestinal barrier can be disrupted by various factors, leading to increased intestinal permeability and allowing for the translocation of pathogens, bacterial endotoxins, such as lipopolysaccharide (LPS), and food‐derived metabolites into the bloodstream [5]. This dysfunction, often termed “leaky gut,” is known to induce chronic inflammation and exert detrimental systemic effects on extraintestinal tissues [6, 7].

Leaky gut generally refers to an increase in intestinal permeability resulting from the disruption of the intestinal barrier; however, “leaky gut syndrome” has not been established as an independent medical diagnosis [8]. On the other hand, some studies define leaky gut as a pathological increase in intestinal permeability [9]. Therefore, in this review, we define leaky gut as a state of pathologically increased intestinal permeability. In leaky gut, paracellular permeability, transcellular permeability, and microbial translocation/endotoxemia may all be increased. Paracellular permeability is altered by disruption of the tight junction (TJ)–dependent pore, leak, and unrestricted pathways, whereas transcellular permeability is enhanced through increased endocytosis and transcytosis of luminal antigens and macromolecules [10, 11]. As barrier dysfunction progresses, microbial products such as LPS can enter the circulation, thereby promoting local and systemic inflammation [12]. Conditions in which increased intestinal permeability is relatively well established include celiac disease, inflammatory bowel disease (IBD), and gastrointestinal graft‐versus‐host disease (GVHD) [13]. In contrast, although the involvement of altered intestinal permeability has been suggested in disorders of gut–brain interaction (DGBI), including irritable bowel syndrome (IBS), and in metabolic dysfunction–associated steatotic liver disease (MASLD), its causal role and pathological significance remain under debate [14, 15].

Furthermore, leaky gut should not be regarded as the same phenomenon in the small and large intestines: In the small intestine, abnormalities in epithelial junctions and absorptive epithelial function are predominant, whereas in the large intestine, disruption of the mucus barrier and increased bacterial contact with the epithelium are more relevant [16]. Thus, when discussing leaky gut, it is essential to specify the target intestinal region.

To date, many in vivo and in vitro studies have explored the molecular mechanisms of intestinal barrier disruption, yet the detailed pathophysiology has not been fully elucidated. This review is aimed at summarizing the current understanding of the components of the intestinal barrier and the systemic consequences of their dysfunction, thereby highlighting the importance of the intestinal barrier as a potential therapeutic target.

## 2. Composition of the Intestinal Barrier

The intestine is composed of many different cell types that interact both physically and functionally to maintain intestinal integrity and homeostasis. The physical barrier, formed by the close adhesion of intestinal epithelial cells, restricts the translocation of harmful substances from the lumen into underlying tissues. The chemical barrier is characterized by the secretion of mucus and antimicrobial peptides (AMPs) into the intestinal lumen, thereby preventing direct contact between epithelial cells and potentially harmful substances or pathogens. The microbiological barrier, established by commensal microbiota, inhibits the colonization and persistence of pathogenic microorganisms within the intestinal lumen. The immunological barrier, comprising immune cells located in the lamina propria beneath the epithelium, contributes to intestinal homeostasis through the production of antimicrobial molecules and modulation of immune responses. These barriers maintain intestinal and systemic homeostasis by preventing the translocation of pathogens and other harmful luminal substances [16].

**Figure 1:**
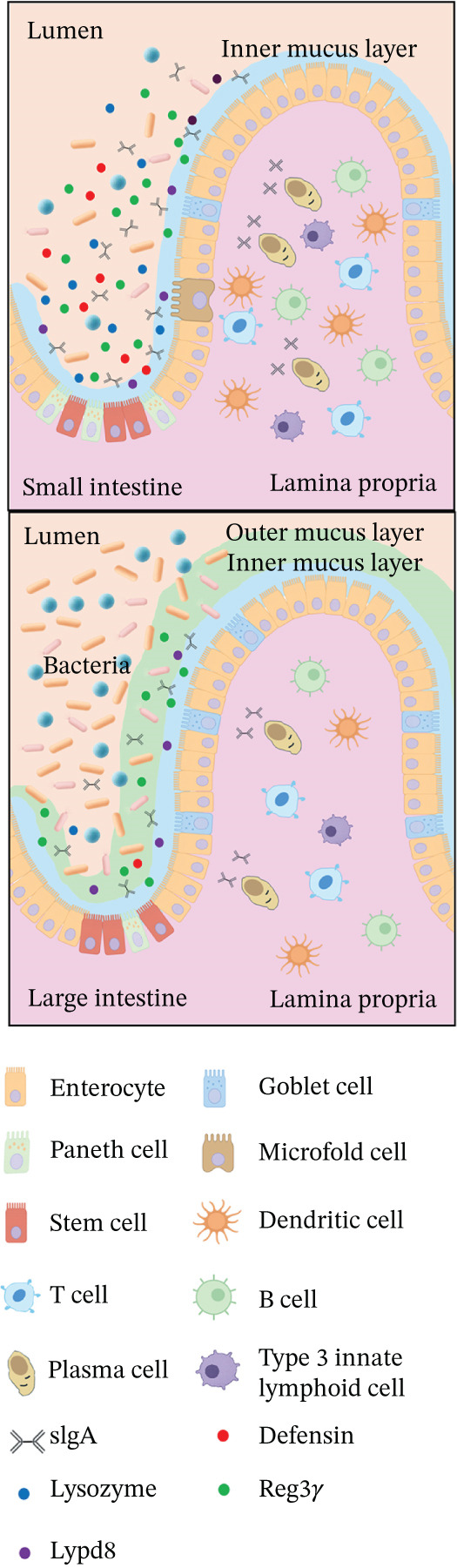
Distinct defense strategies in the small and large intestines. The small and large intestines differ in the composition of villus‐associated cell populations, as well as in the type and abundance of the gut microbiota, and therefore rely on distinct defense strategies. In the small intestine, goblet cells are relatively sparse and the mucus layer is single layered (inner mucus layer only). Paneth cells are abundant and produce a wide range of antimicrobial factors that regulate the intestinal microbiota. The bacterial load is also lower than that in the large intestine. Thus, barrier function is maintained mainly by preventing commensal bacteria from approaching the epithelium through antimicrobial activity. In the colon, goblet cells are abundant, and the mucus layer consists of two layers: an inner adherent layer and an outer secreted layer. In contrast, Paneth cells are less numerous and antimicrobial factor production is lower than in the small intestine. The colon also harbors a more diverse and abundant microbiota. Accordingly, barrier function is maintained primarily by the two‐layered mucus structure, which spatially segregates commensal bacteria from the epithelium. Among the intestinal epithelial cells, stem cells and Paneth cells are located in the crypts, whereas goblet cells are interspersed among enterocytes along the villus axis toward the apical region. Microfold cells are distributed among enterocytes from the crypt region to the apical side of the small intestinal epithelium. Various immune cells, including dendritic cells, T cells, B cells, plasma cells, and Type 3 innate lymphoid cells, are present in the lamina propria. The intestinal lumen contains bacteria and various antimicrobial substances, including sIgA, defensins, lysozyme, Reg3*γ*, and Lypd8. Because some sIgA is produced by plasma cells, it is also present in the lamina propria.

As mentioned above, the small and large intestines differ in the principal components of their barrier systems. Although the intestinal barriers of both regions comprise physical, chemical, microbiological, and immunological components, they differ in cellular composition, mucus structure, and the characteristics of the gut microbiota. Consequently, the small and large intestines utilize distinct defense strategies. In the small intestine, defense is driven predominantly by antimicrobial factors derived from Paneth cells, whereas in the large intestine, spatial segregation of bacteria from the epithelium by a bilayered mucus barrier plays a more central role (Figure 1).

### 2.1. Physical Barrier

**Figure 2:**
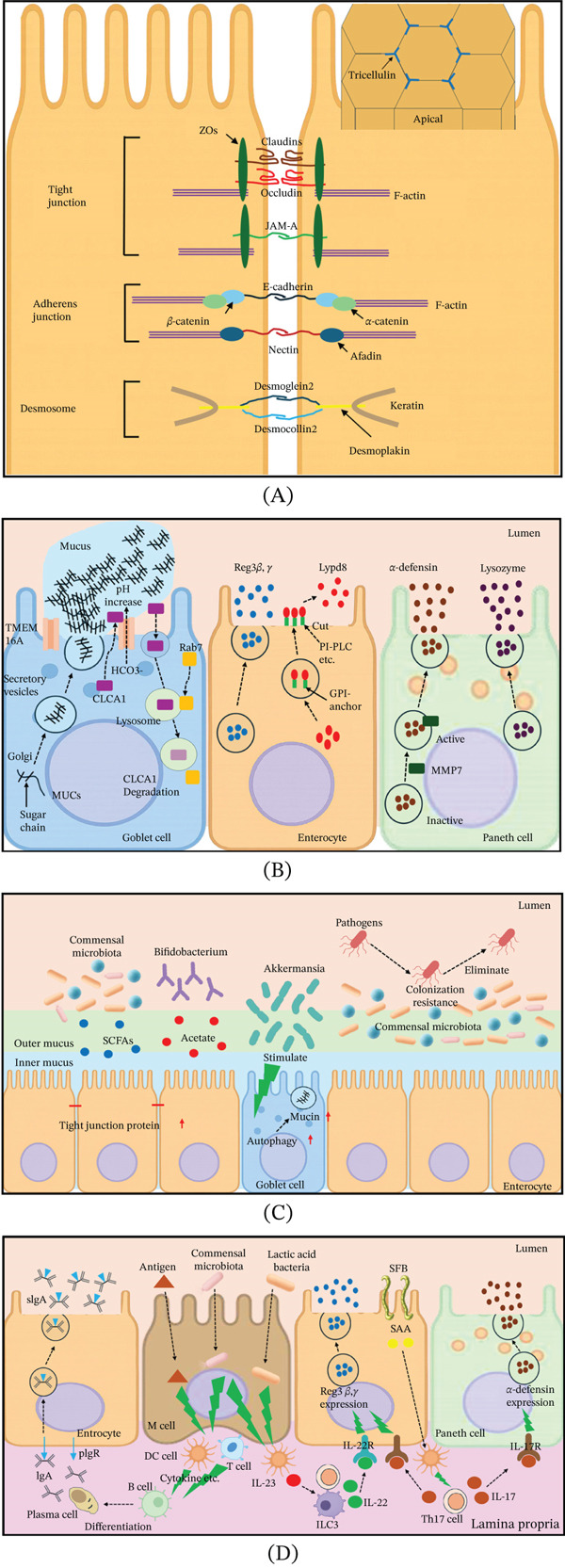
The intestinal barrier is composed of four components. (A) Physical barrier: composed of intercellular junctions between intestinal epithelial cells, including tight junctions, adherens junctions, and desmosomes. In tight junctions, claudins, occludin, and JAM proteins form homophilic interactions between adjacent cells and are linked intracellularly to ZO proteins, which connect to F‐actin to stabilize cell–cell adhesion. In adherens junctions, E‐cadherin binds homophilically between neighboring cells and is associated intracellularly with *β*‐catenin and *α*‐catenin, which connect other scaffold proteins and F‐actin bundles. Desmosomes are formed by desmoglein‐2 and desmocollin‐2, which bind homophilically on adjacent cells and are linked intracellularly to desmoplakin, which anchors the complex to keratin intermediate filaments. Tricellulin localizes to tricellular contacts at the apical region of epithelial cells and helps maintain adhesion where three cells meet. (B) Chemical barrier: composed of mucus derived from goblet cells and antimicrobial substances released by Paneth cells and enterocytes. In goblet cells, mucin proteins glycosylated in the Golgi apparatus are transported to the apical surface via the secretory vesicles and secreted into the lumen. CLCA1 stabilizes TMEM16A, which promotes luminal HCO3^−^ transport and loosens the mucin gel structure, thereby increasing its fluidity. After endocytic uptake, CLCA1 is transported to lysosomes via Rab7 and degraded. Paneth cells mainly secrete *α*‐defensins and lysozyme. *α*‐defensins are synthesized as inactive precursors, activated by MMP‐7 during trafficking in the secretory granules toward the apical surface, and then secreted into the lumen. Lysozyme is likewise transported to the apical surface in the secretory granules after synthesis and released into the lumen. Enterocytes mainly secrete Reg3 proteins (Reg3*β* and Reg3*γ*) and Lypd8. After synthesis, Reg3 proteins are transported in the secretory vesicles to the apical surface and secreted into the lumen. Lypd8 is synthesized, modified with a GPI anchor, trafficked in the secretory vesicles to the apical surface, and secreted into the lumen. (C) Microbiological barrier: Resident commensal microbiota provide colonization resistance against invading pathogens. In addition, microbiota‐derived metabolites reinforce the physical barrier of the intestinal epithelium. Commensal microbiota, including *Bifidobacterium*, enhance the expression of tight junction proteins in epithelial cells through the production of short‐chain fatty acids, including acetate. Furthermore, *Akkermansia* stimulates goblet cells, thereby promoting intracellular autophagy and increasing mucin expression. (D) Immunological barrier: Immune cells in the lamina propria respond to luminal stimuli and regulate immune cell differentiation and epithelial antimicrobial programs. Luminal commensal microbiota, including lactic acid bacteria, and food antigens are sampled by M cells, leading to activation of T cells and dendritic cells, which promote B‐cell differentiation into IgA‐producing plasma cells. IgA binds pIgR on enterocytes, is transcytosed in the secretory vesicles, and is released apically as sIgA. In parallel, IL‐23 stimulates ILC3s and Th17 cells to produce IL‐22, which enhances Reg3 expression in enterocytes. SFB stimulate epithelial SAA production, and SAA activates lamina propria dendritic cells to produce cytokines that drive Th17 differentiation and IL‐17 production. IL‐17 acts on enterocytes and Paneth cells to increase Reg3 and *α*‐defensin expression. JAM; junctional adhesion molecule, ZO; zonula occludens, TMEM16A; transmembrane Member 16A, CLCA1; chloride channel Accessory 1, Rab7; Ras‐related protein in Brain 7, Reg3; regenerating islet‐derived Protein 3, Lypd8; LY6/PLAUR domain‐containing 8, M cell; microfold cell, DC cell; dendritic cell, sIgA; secretory immunoglobulin A, pIgR; polymeric immunoglobulin receptor, ILC3; Type 3 innate lymphoid cell, IL‐22R; interleukin‐22 receptor, IL‐17R; interleukin‐17 receptor, SFB; segmented filamentous bacteria.

The intestinal epithelium is composed of various cell types, four of which play key roles in maintaining the intestinal barrier: enterocytes, goblet cells, Paneth cells, and microfold (M) cells [17]. Additionally, other cell types, such as intestinal stem cells, enteroendocrine cells, including L and K cells, and tuft cells, also contribute to intestinal and systemic homeostasis [18, 19]. The physical barrier formed by these cells consists of three principal junctional complexes: TJs, adherens junctions (AJs), and desmosomes (Figure 2A) [3, 20].

TJ is composed of transmembrane proteins, including claudins, occludin, junctional adhesion molecules (JAMs), and tricellulin, and intracellular scaffolding proteins, such as zonula occludens (ZO) proteins and cingulin [21]. Claudins assemble into strands at TJ [3]. In mammals, the claudin family comprises at least 27 members with tissue‐specific expression patterns [22]. The claudins expressed in the human intestine (duodenum to rectum) include claudin‐1, ‐2, ‐3, ‐4, ‐5, ‐7, ‐8, ‐12, and ‐15, whereas the mouse intestine expresses claudin‐1, ‐2, ‐3, ‐4, ‐5, ‐7, ‐8, ‐12, ‐13, and ‐15 [23]. Although most claudins, such as claudin‐1, ‐3, ‐4, ‐5, ‐7, and ‐19, are essential for the barrier function, claudin‐2 and ‐15 are known as “channel‐forming” claudins that form selective pores for ions and water, facilitating their absorption [24]. Claudin‐3 or claudin‐7 knockout mice exhibit an increase in intestinal permeability, indicating their importance in maintaining the intestinal barrier [25, 26]. Upregulation of claudin‐2 in the gut increases intestinal permeability and exacerbates experimental colitis. Consistently, claudin‐2 knockout attenuates disease severity, indicating that claudin‐2 is a critical regulator of intestinal barrier function [27].

Occludin was the first identified TJ protein [28] and was initially considered crucial for normal barrier function [29]. However, occludin knockout cells can develop morphologically normal TJs [30], and occludin knockout mice show no defects in intestinal barrier function [31]. Thus, occludin appears to be dispensable for the structural and functional integrity of TJs in the intestine. Nevertheless, the occludin knockout mice display extraintestinal pathologies, such as chronic gastric inflammation, suggesting that occludin may influence the TJ composition or function in other tissues [31]. The plasma membrane localization of occludin is regulated by posttranslational modifications. The membrane‐bound occludin is highly phosphorylated at serine/threonine residues and unphosphorylated at tyrosine residues. In vitro studies have shown that phosphorylation of tyrosine residues in the C‐terminal domain of occludin is mediated by c‐Src tyrosine kinase and this phosphorylation leads to the disruption of its binding to ZO‐1, a scaffold protein in TJs [32, 33].

The JAMs, including JAM‐A, B, and C, are other TJ components belonging to the immunoglobulin superfamily [34, 35]. Mice with a colon epithelial cell–specific knockout of JAM‐A exhibit increased intestinal permeability despite having a normal epithelial architecture [36]. Furthermore, the loss of JAM‐A leads to increased expression of claudin‐10 and ‐15. As these are pore‐forming claudins, their upregulation may account for the increased permeability caused by JAM‐A deficiency [36]. Under inflammatory conditions, JAM‐A can be phosphorylated by Yes‐1, a Src family tyrosine kinase, which causes its dissociation from Rap2c, a Ras family small GTPase that is reported to promote cell‐to‐cell adhesion and eventually increases intestinal permeability [37]. Thus, JAM‐A is required for the maintenance of barrier function and is involved in the regulation of the expression levels of other TJ proteins.

Tricellulin (marvelD2) is a TJ protein that localizes specifically to tricellular contacts, where three cells meet [38, 39]. It has been reported that tricellulin is recruited to the plasma membrane by angulin family members (angulin‐1, ‐2, or ‐3) [40]. Knockdown of tricellulin in cultured mouse epithelial cells weakens tricellular contacts and also alters occludin distribution and overall TJ morphology [38]. Moreover, tricellulin knockdown results in a decrease in transepithelial electrical resistance (TEER) and an increase in paracellular permeability, indicating its critical role in barrier function [41].

ZO proteins, ZO‐1, ZO‐2, and ZO‐3, belong to the membrane‐associated guanylate kinase (MAGUK) superfamily and share several common structures: three PDZ domains, one Src Homology 3 (SH3) domain, and one guanylate kinase (GUK) domain [42]. ZO‐1 links transmembrane TJ proteins (claudins, occludin, and JAMs) to actin cytoskeleton, thereby anchoring the TJ complex to maintain barrier function [43]. Phosphorylation of a tyrosine residue in the PDZ1 domain of ZO‐1 has been reported to decrease its binding affinity for claudins, which perturbs TJ organization [44]. In vitro, the loss of either ZO‐1 or ZO‐2 alone in MDCK II epithelial cells does not affect TJ structure, but the combined loss of both proteins leads to severe structural defects of the TJ, suggesting they have overlapping functions [45]. However, mice lacking only ZO‐1 exhibit increased intestinal permeability, indicating a nonredundant and essential role of ZO‐1 in intestinal barrier function in vivo [46]. In addition to its role in TJ function, ZO‐1 has also been implicated in regulating epithelial cell proliferation [46]. Thus, ZO‐1 is a multifunctional TJ protein crucial for intestinal homeostasis.

AJs are located just basal to TJs and constitute another key component of the physical barrier [47, 48]. They are composed of adhesion molecules, cadherins and nectins, and intracellular scaffolding proteins, such as catenins and afadin, which link cell adhesion molecules to cytoskeletons [49–51]. The cadherin superfamily is classified into classic and nonclassic cadherins. E‐cadherin, a classic cadherin, is abundantly expressed in intestinal epithelial cells, and its cytoplasmic region binds to *β*‐catenin and p120‐catenin [47]. Because *β*‐catenin interacts with actin cytoskeleton via *α*‐catenin, catenins stabilize the cadherin‐mediated cell adhesion complex [52, 53]. Mice with an intestinal epithelial cell–specific deletion of E‐cadherin exhibit severe intestinal morphogenesis defects and die within 24 h after birth [54]. Furthermore, tamoxifen‐induced deletion of E‐cadherin in adult mouse intestinal epithelial cells causes AJ disruption, a significant increase in intestinal permeability, and hemorrhagic diarrhea [55]. Interestingly, occludin localization remains normal in these mice, suggesting that the loss of AJ integrity may not necessarily disrupt TJ structure [56]. These data indicate that E‐cadherin is essential for maintaining the intestinal barrier and overall organismal homeostasis.

Nectins are immunoglobulin‐like cell adhesion molecules that bind to an actin‐binding protein, afadin [57]. It has been reported that nectins are localized at AJ prior to cadherins and recruit the cadherin–catenin complex to the AJ [58]. In fact, nectins are reported to play a role in establishing barrier functions by recruiting cadherins to the plasma membrane [59]. Intestinal epithelial cell–specific afadin knockout mice exhibit increased intestinal permeability, whereas deletion of nectin‐2 or nectin‐3 does not alter permeability, indicating that afadin is critical for maintaining the intestinal barrier [60].

Desmosomes are intercellular adhesion structures located at the most basolateral position of the apical junctional complex. The primary adhesion molecules in desmosomes are desmosomal cadherins, which include desmogleins (Dsgs) and desmocollins (Dscs) [61, 62]. In intestinal epithelial cells, the main isoforms expressed are Dsg2 and Dsc2 [63]. Dsg and Dsc interact with desmoplakin, which in turn binds keratin intermediate filaments, thereby anchoring the desmosomal complex [64]. Mice deficient in Dsg2 exhibit altered desmosome structure, decreased expression of claudin‐1, and increased intestinal permeability [65]. In contrast, mice deficient in Dsc2 do not show increased susceptibility to the barrier dysfunction induced by dextran sodium sulfate (DSS) administration, suggesting that Dsc2 is less critical for intestinal barrier function (Table 1) [66].

**Table 1:** Major proteins involved in the intestinal physical barrier.

Protein	Phenotype	Function
Claudins	Claudin‐3 or claudin‐7 deficiency increases intestinal permeability.	Major transmembrane components of tight junction strands; regulate barrier sealing.
	Claudin‐2 upregulation enhances permeability and worsens colitis, whereas claudin‐2 deficiency attenuates disease severity.	Major transmembrane components of tight junction strands; regulate selective ion/water permeability.
Occludin	Occludin deficiency does not cause obvious intestinal barrier defects, although extraintestinal abnormalities are observed.	Tight junction–associated transmembrane protein that modulates tight junction organization and signaling rather than serving as an essential structural component in the intestine.
JAM‐A	Epithelial JAM‐A deficiency increases intestinal permeability and is associated with increased claudin‐10 and claudin‐15 expression.	Tight junction adhesion molecule required for barrier maintenance and regulation of other tight junction proteins.
Tricellulin	Tricellulin knockdown decreases transepithelial electrical resistance and increases paracellular permeability.	Specialized tight junction protein at tricellular contacts that maintains barrier integrity where three cells meet.
Angulin‐1/‐2/‐3	Not specifically described in the attached text.	Recruit tricellulin to tricellular contacts and support tricellular tight junction organization.
ZO‐1	ZO‐1 deficiency increases intestinal permeability	Scaffold protein that links tight junction transmembrane proteins to the actin cytoskeleton and stabilizes the junctional complex.
ZO‐2	Loss alone does not severely affect tight junction structure, but combined loss with ZO‐1 causes marked defects in vitro.	Scaffold protein with overlapping functions with ZO‐1 in tight junction organization.
ZO‐3	Not specifically described in the attached text.	Tight junction–associated scaffold protein.
Cingulin	Not specifically described in the attached text.	Cytoplasmic tight junction–associated scaffold protein.
E‐cadherin	Intestinal epithelial deletion causes severe morphogenesis defects, increased permeability, and hemorrhagic diarrhea.	Core adherens junction adhesion molecule essential for epithelial integrity and barrier maintenance.
*β*‐catenin	Not specifically described in the attached text.	Intracellular adaptor linking E‐cadherin to *α*‐catenin.
*α*‐catenin	Not specifically described in the attached text.	Links the cadherin–catenin complex to the actin cytoskeleton.
p120‐catenin	Not specifically described in the attached text.	Stabilizes the cadherin adhesion complex.
Nectin‐2/nectin‐3	Deletion of nectin‐2 or nectin‐3 does not alter intestinal permeability.	Immunoglobulin‐like adhesion molecules that help recruit the cadherin–catenin complex during adherens junction formation.
Afadin	Intestinal epithelial afadin deficiency increases intestinal permeability	Actin‐binding scaffold protein essential for adherens junction–mediated barrier maintenance.
Desmoglein‐2 (Dsg2)	Dsg2 deficiency alters desmosome structure, decreases claudin‐1 expression, and increases intestinal permeability.	Major desmosomal cadherin that supports strong epithelial adhesion.
Desmocollin‐2 (Dsc2)	Dsc2 deficiency does not increase susceptibility to DSS‐induced barrier dysfunction.	Desmosomal cadherin with a less critical role than Dsg2 in intestinal barrier maintenance.
Desmoplakin	Not specifically described in the attached text.	Anchors the desmosomal complex to keratin intermediate filaments.
Keratin	Not specifically described in the attached text.	Intermediate filament that provides mechanical strength to epithelial cells.

### 2.2. Chemical Barrier

The chemical barrier physically separates luminal microbes from the intestinal epithelium. It is primarily composed of mucus, secreted by goblet cells, and AMPs secreted by Paneth cells, which collectively prevent commensal bacteria and pathogens from damaging the host (Figure 2B).

The main structural component of mucus is mucin, a large glycoprotein. The core proteins are encoded by a family of genes designated *MUC*, which comprises at least 22 members (*MUC1–MUC22*) with distinct tissue and cell‐type expression patterns [67]. Humans express five membrane‐bound mucins: MUC3, MUC4, MUC12, MUC13, and MUC17, with MUC3 being abundant in the duodenum, MUC17 in the small intestine, and MUC12 in the large intestine [68, 69].

The intestinal mucus layer is organized differently along the gastrointestinal tract, a single layer in the small intestine and a distinct two‐layered structure in the large intestine [70]. The inner colonic mucus layer is firmly attached to the epithelium and is composed of membrane‐bound transmembrane mucins, whereas the outer mucus layer contains gel‐forming mucins, a major component of which is goblet cell‐derived MUC2 in both humans and mice [71]. This outer mucus layer keeps bacteria at a safe distance from the epithelium while simultaneously providing a habitat for specific intestinal bacteria [72–74].

The mucus layer is not static but continuously turned over. This dynamic process is regulated by a zinc‐dependent metalloproteinase, chloride channel Accessory 1 (CLCA1). Secreted CLCA1 stabilizes cell‐surface calcium‐activated anion channels, such as transmembrane Protein 16A (TMEM16A) [75]. This stabilization facilitates the transport of large quantities of bicarbonate ions (HCO3^−^) into the lumen [76]. The resulting local alkalinization loosens the mucus gel structure, reduces its density, and promotes the flow of newly secreted mucus from the inner to the outer layer [77]. The secretion of CLCA1 is indirectly regulated by Rab7, a Rab family small GTPase that is involved in late endosomal transport and lysosomal degradation pathways [78–80]. In Rab7‐deficient mice, the lysosomal degradation of CLCA1 is impaired and thereby luminal CLCA1 levels are increased. This induces excessive mucus fluidization, which decreases barrier integrity and allows bacteria to approach the epithelium [81]. Thus, the mucus plays an important role in the intestinal barrier and is regulated by various proteins.

AMPs function to inhibit microbial growth and are primarily secreted by Paneth cells. Major classes of intestinal AMPs include defensins, lysozyme, and regenerating (Reg) islet‐derived family proteins [82]. Paneth cells are abundant in the human small intestine, cecum, and ascending colon but are sparse in the descending colon and rectum. In mice, they are found predominantly in the small intestine [83].

The defensin family is divided into *α*‐, *β*‐, and *θ*‐defensin. *β*‐defensin is expressed in various epithelial cells, and their production is induced by infectious stimuli as part of the innate immune response [84]. *θ*‐defensin is not expressed in humans, although its expression in monocytes of Old World monkeys has been reported [85]. *α*‐defensin is the most studied component concerning the intestinal barrier. These basic peptides (18–45 amino acids) have potent bactericidal effects against pathogens while largely sparing commensal bacteria [86]. In humans, Paneth cells secrete *α*‐defensin 5 (DEFA5) and DEFA6 [87]. In mice, *α*‐defensins are known as cryptdins, and six isoforms have been identified. Among them, cryptdin‐4 has the most potent antibacterial activity [88, 89]. *α*‐defensins are secreted as inactive propeptides and require proteolytic cleavage by matrix Metalloproteinase 7 (MMP‐7) for activation [90]. Consequently, MMP‐7–deficient mice are unable to activate *α*‐defensins and show higher mortality rates than wild‐type mice following oral infection with *Salmonella* [91].

Lysozyme is a glycosidase that targets gram‐positive bacteria by hydrolyzing the *β*‐(1,4)‐glycosidic bonds between N‐acetylmuramic acid and N‐acetylglucosamine within their peptidoglycan cell walls [92]. The cell wall of gram‐positive bacteria is mainly composed of peptidoglycan alone, whereas gram‐negative bacteria have an outer membrane of LPS outside the peptidoglycan [93], and therefore, lysozyme affects only gram‐positive bacteria. In mice, Lysozyme 1 (also termed lysozyme P) is expressed in the intestinal epithelium, whereas Lysozyme 2 (lysozyme M) is expressed primarily in immune cells. Paneth cell–specific Lysozyme 1 knockout mice display an increased relative abundance of the gram‐positive bacterium *Ruminococcus gnavus* [94]. Lysozyme modulates the intestinal barrier: Elevating luminal lysozyme increases paracellular permeability, whereas Paneth cell–specific lysozyme deletion reduces it [94, 95]. Thus, enhanced intestinal lysozyme secretion may compromise barrier integrity.

Reg family comprises C‐type lectins (calcium‐dependent glycan‐binding proteins). Among seven subtypes of Reg proteins in mice (Reg1, Reg2, Reg3*α*, Reg3*β*, Reg3*δ*, Reg3*γ*, and Reg4), Reg3s are highly expressed in the intestine [96–99]. These proteins have distinct targets: Reg3*β* binds to the LPS of gram‐negative bacteria, whereas Reg3*γ* binds to the peptidoglycan of gram‐positive bacteria [100, 101]. Reg3*γ* secretion is stimulated by Type 3 innate lymphoid cells (ILC3)–mediated interleukin‐22 (IL‐22) [102]. Lactic acid bacteria administered to mice stimulate IL‐23 secretion from dendritic cells, which induces IL‐22 production from ILC3 [103]. In Reg3*γ*‐deficient mice, the intestinal gram‐positive bacteria are increased, which in turn activates immune cells in the intestines [101]. In these mice, the spatial segregation between the microbiota and the epithelium is reduced by approximately 50 *μ*m in Reg3*γ*‐deficient mice. This leads to increased bacterial contact with epithelial cells and subsequent aberrant activation of the intestinal immune system.

LY6/PLAUR domain‐containing 8 (Lypd8) is a glycosylphosphatidylinositol (GPI)–anchored protein expressed in colon epithelial cells. After synthesis, Lypd8 undergoes GPI‐anchor attachment and glycosylation, and is subsequently transported in secretory vesicles to the cell surface, where it is tethered to the outer leaflet of the plasma membrane. Subsequently, the GPI‐anchor is thought to be cleaved potentially by phosphatidylinositol‐specific phospholipase C, thereby releasing Lypd8 into the lumen [104]. Lypd8 may prevent bacterial invasion into the inner mucus layer by binding to bacterial flagella and suppressing their motility [105]. Lypd8‐deficient mice exhibit increased contact between flagellated bacteria and the colonic epithelium, leading to heightened susceptibility to experimental colitis (Table 2) [106].

**Table 2:** Major proteins involved in the intestinal chemical barrier.

Protein	Phenotype	Function
Membrane‐bound mucins (MUC3, MUC4, MUC12, MUC13, and MUC17)	Exhibit region‐specific expression in the human intestine; MUC3 is abundant in the duodenum, MUC17 in the small intestine, and MUC12 in the large intestine.	Contribute to the epithelial surface glycocalyx and the attached/inner mucus layer, thereby protecting the mucosal surface from luminal microbes.
MUC2	The major goblet cell‐derived gel‐forming mucin in the outer colonic mucus layer in both humans and mice.	Forms the structural backbone of the mucus barrier and spatially separates luminal bacteria from the epithelium while also providing a habitat for selected commensals.
CLCA1	A zinc‐dependent metalloproteinase secreted into the lumen; Rab7 deficiency impairs its lysosomal degradation, increases luminal CLCA1, and causes excessive mucus fluidization with reduced barrier integrity.	Regulates mucus turnover and fluidization by stabilizing TMEM16A and promoting mucus expansion and movement from the inner to the outer layer.
TMEM16A	A calcium‐activated anion channel stabilized by CLCA1.	Facilitates bicarbonate (HCO3^−^) transport into the lumen, thereby loosening the mucus gel structure and increasing mucus fluidity.
Rab7	A small GTPase involved in late endosomal transport and lysosomal degradation; its deficiency increases luminal CLCA1 and compromises barrier integrity.	Indirectly regulates mucus homeostasis by controlling CLCA1 trafficking and degradation.
*α*‐Defensins	Basic peptides (18–45 amino acids) secreted as inactive propeptides; display potent bactericidal activity against pathogens while largely sparing commensals.	Major Paneth cell–derived antimicrobial peptides that limit microbial overgrowth and protect the host epithelium.
Cryptdins	Murine alpha‐defensin isoforms; cryptdin‐4 exhibits the strongest antibacterial activity.	Represent the major alpha‐defensin system in mice and contribute to antimicrobial control in the intestinal lumen.
MMP‐7	Required for proteolytic activation of alpha‐defensins; MMP‐7–deficient mice fail to activate alpha‐defensins and show higher mortality after oral *Salmonella* infection.	Processes inactive pro‐alpha‐defensins into their active bactericidal forms.
Lysozyme 1 (lysozyme P)	Expressed in the intestinal epithelium in mice; Paneth cell–specific deletion increases the relative abundance of *Ruminococcus gnavus*. Increased luminal lysozyme elevates paracellular permeability, whereas Paneth cell–specific deletion reduces it.	Hydrolyzes peptidoglycan in gram‐positive bacteria and modulates microbial composition and barrier properties.
Lysozyme 2 (lysozyme M)	Expressed primarily in immune cells in mice rather than in the intestinal epithelium.	Functions as a glycosidase targeting bacterial cell‐wall peptidoglycan.
Reg3*β*	A Reg family C‐type lectin highly expressed in the intestine; binds lipopolysaccharide (LPS) of gram‐negative bacteria.	Acts as an antimicrobial lectin targeting gram‐negative bacteria.
Reg3*γ*	Binds peptidoglycan of gram‐positive bacteria and is induced by ILC3‐derived IL‐22. Reg3gamma deficiency increases intestinal gram‐positive bacteria, activates immune cells, and reduces microbiota–epithelium segregation by approximately 50 *μ*m.	Maintains spatial segregation between microbiota and the epithelium and contributes to antimicrobial defense against gram‐positive bacteria.
IL‐22	Produced downstream of IL‐23–mediated activation of ILC3; enhances Reg3 expression in enterocytes.	Links immune signaling to the chemical barrier by inducing antimicrobial proteins in the epithelium.
Lypd8	A GPI‐anchored protein expressed by colon epithelial cells; thought to be released into the lumen after cleavage of its GPI anchor. Lypd8 deficiency increases contact of flagellated bacteria with the colonic epithelium and heightens susceptibility to experimental colitis.	Binds bacterial flagella and suppresses bacterial motility, thereby preventing invasion of the inner mucus layer.

### 2.3. Microbiological Barrier

Although the intestinal microbiota comprises diverse microorganisms, including fungi, archaea, and viruses, this review will focus specifically on the role of bacteria in forming the microbiological barrier (Figure 2C). The human intestinal microbiota consists of approximately 40 trillion bacteria from 500 to 1000 species [107, 108]. Colonization begins at birth, and the composition of the microbiota evolves throughout life: It is dominated by *Bifidobacterium* in infancy, diversifies during childhood, stabilizes in adulthood, and shifts again in old age with an increase of *Bacteroides*. This composition is also influenced by environmental factors and diet [109–111].

The intestinal microbiota maintains a symbiotic relationship with the host. In exchange for a nutrient‐rich habitat, these bacteria metabolize dietary components, such as complex polysaccharides, into host‐absorbable metabolites [112]. Beyond the metabolic support, a key aspect of this symbiosis is the microbiological barrier, a phenomenon also known as “colonization resistance,” where the commensal microbiota actively prevents the colonization of invading pathogens. For example, following oral administration of *Citrobacter rodentium*, specific pathogen‐free (SPF) mice with a conventional microbiota resist colonization, whereas germ‐free (GF) mice lacking a microbiota develop severe enteritis [113]. Similarly, colonization by *Clostridioides difficile* (formerly *Clostridium difficile*) is facilitated by reduced gut microbial diversity and altered community structure, characterized by the expansion of Lactobacillaceae and Enterobacteriaceae and decline in *Bacteroides* and *Bifidobacterium* [114]. Moreover, vancomycin‐resistant *Enterococcus* (VRE), which can increase in the gut after antimicrobial treatment, has been reported to be prevented from colonizing the intestine when a microbiota containing *Barnesiella* is present. In humans, analyses of patients undergoing hematopoietic stem cell transplantation have shown that the presence of *Barnesiella* in the gut inhibits VRE colonization and contributes to suppression of bacteremia [115].

The intestinal microbiota contributes to barrier integrity through both direct and indirect mechanisms [116]. In addition to directly competing with pathogens, the microbiota indirectly reinforces other barriers. For instance, commensal bacteria stimulate the secretion of AMPs like Reg3*γ*, bolstering the chemical barrier [117]. Microbial metabolites, such as short‐chain fatty acids (SCFAs) (e.g., acetate, propionate, and butyrate), are also known to enhance the physical barrier by upregulating the expression of TJ proteins [118, 119]. Specifically, acetate produced by *Bifidobacterium* has been shown to protect against toxin invasion from enterohemorrhagic *Escherichia coli* O157:H7 by strengthening the barrier [120]. Furthermore, propionic acid produced by commensal bacteria, including members of the genus *Bacteroides*, inhibits intestinal colonization by *Salmonella Typhimurium* [121]. Butyrate and butyrate‐producing bacteria reinforce the microbiological barrier by enhancing colonization resistance against enteric pathogens, inducing AMPs such as Reg3*γ* and *β*‐defensins, and suppressing pathogen virulence programs [122–124]. Moreover, certain commensals like *Akkermansia muciniphila*, a mucin‐degrading specialist, reinforce the chemical barrier by stimulating goblet cell autophagy, which increases mucin secretion and goblet cell number [125, 126].

Intestinal bacteria not only promote the secretion of Reg3*γ* and mucins, but also enhance immunoglobulin A (IgA) production, which contributes to the immunological barrier described below. Specifically, colonization of GF mice with intestinal bacteria induces an IgA response directed against the commensal microbiota. In other words, the presence of intestinal bacteria triggers IgA production in the gut [127]. Therefore, the microbiota is an integral component of host defense, acting not only as a barrier itself but also as a key regulator of the physical, chemical, and immunological barriers.

### 2.4. Immunological Barrier

Beneath the intestinal epithelium lies the lamina propria, a key site for the immunological barrier. Within this layer, immune cells secrete immunoglobulins, predominantly IgA, which contributes to regulating the intestinal microbiota and eliminating pathogens. In addition, recent studies have shown that cytokines produced by immune cells, such as IL‐17 and IL‐22, contribute to the maintenance of the intestinal barrier by acting on intestinal epithelial cells (Figure 2D).

Although IgA is present in both the blood and mucosal surfaces, the dimeric secretory form (sIgA), which is mainly found at mucosal surfaces, is particularly important for regulating the intestinal microbiota [128]. The production of IgA is initiated in gut‐associated lymphoid tissues, such as Peyer′s patches. In Peyer′s patches, M cells transport luminal antigens, which are mainly derived from the intestinal microbiota, to underlying dendritic cells. These dendritic cells then stimulate B cells through antigen presentation and cytokine signaling, inducing their differentiation into IgA‐producing plasma cells [129]. B cells can also be stimulated through antigen presentation by T cells, a process that generates high‐affinity IgA antibodies that specifically target pathogenic or opportunistic bacteria [128, 130].

Once produced, dimeric IgA is recognized by the polymeric immunoglobulin receptor (pIgR) on the basolateral surface of intestinal epithelial cells. It is then transported across the epithelial cell via transcytosis and secreted into the lumen as sIgA [131]. The importance of this system is highlighted in knockout mouse models. IgA‐deficient mice exhibit increased levels of inflammatory cytokines (e.g., IL‐17), ileal inflammation, and gut dysbiosis [132]. Similarly, pIgR‐deficient mice, which cannot secrete sIgA into the intestinal lumen, show increased susceptibility to *Salmonella* infection [133, 134]. Therefore, luminal sIgA is essential for maintaining the intestinal barrier through immune responses and the regulation of the microbiota.

In the intestine, IL‐17 is produced primarily by Th17 cells, *γδ* T cells, and ILC3s, and its production is induced by microbiota‐ and pathogen‐derived signals mediated through cytokines, such as IL‐6, IL‐1*β*, TGF‐*β*, and IL‐23. Notably, segmented filamentous bacteria (SFB) are well recognized as representative commensals that strongly promote Th17 cell differentiation in the small intestine [135]. Stimulation by SFB induces serum amyloid A (SAA) production in enterocytes, which in turn acts on dendritic cells to promote the production of cytokines that drive Th17 cell differentiation [136]. IL‐17 acts on intestinal epithelial cells through the IL‐17 receptor complex (IL‐17RA/IL‐17RC) and contributes to intestinal barrier homeostasis by promoting the expression of antimicrobial molecules, such as Reg3*γ* and *β*‐defensins, maintaining Paneth cell function, regulating TJ‐associated proteins, and enhancing epithelial repair responses [137]. Indeed, in DSS‐induced colitis models, IL‐17A has been shown to preserve epithelial barrier integrity by regulating the subcellular localization of occludin, thereby limiting excessive intestinal permeability [138]. In addition, IL‐17 signaling has been implicated in the differentiation and maintenance of Paneth cells, and recent studies have demonstrated that IL‐17RA signaling within the intestinal epithelial lineage is required to preserve Paneth cell abundance and antimicrobial defense programs [139]. Although moderate IL‐17 signaling exerts protective effects, excessive or chronic activation may exacerbate inflammation. Thus, in the gut, IL‐17 should be regarded not only as a proinflammatory cytokine but also as a critical regulator of mucosal defense and barrier maintenance [140].

Intestinal IL‐22 is produced primarily by ILC3s and Th17/Th22 cells and plays a pivotal role in maintaining the intestinal barrier through its actions on interleukin‐22 receptors (IL‐22R) expressed by intestinal epithelial cells [141, 142]. IL‐22 enhances host defense by inducing antimicrobial molecules such as Reg3*β* and Reg3*γ*, while also promoting epithelial repair and mucosal homeostasis, thereby limiting excessive bacterial contact with the epithelium [143]. In addition, IL‐22 signaling contributes to goblet/progenitor cell programs and mucin‐related pathways involved in barrier protection and regeneration [144]. However, although appropriate IL‐22 signaling is protective, excessive or persistent activation may also contribute to pathogenesis [142].

### 2.5. The Relationships Among the Four Barriers

The intestinal barrier should be regarded not as a collection of separate physical, chemical, microbiological, and immunological barriers, but rather as an integrated defense system in which each component mutually regulates the others. Consequently, disruption of any single layer can initiate a cascade of impairments across the other components. For instance, when the physical barrier is disrupted and epithelial permeability increases, the influx of bacterial components and antigens into the mucosa is enhanced, leading to activation of the immunological barrier. This immune response, characterized by the release of inflammatory cytokines, can further impair TJ function, while secondarily affecting mucus secretion and the intestinal microbiota [3].

Meanwhile, when the chemical barrier is impaired, the spatial segregation between bacteria and the epithelium—normally maintained by the MUC2‐rich mucus layer and antimicrobial molecules such as RegIII*γ*—is lost. This leads to the disruption of the microbiological barrier and excessive activation of the immune cells [101, 145]. In addition, disruption of the microbiological barrier—specifically dysbiosis and a reduction in beneficial metabolites such as SCFAs—simultaneously destabilizes the physical, chemical, and immunological barriers by weakening mucus layer formation, antimicrobial molecule expression, epithelial junction maintenance, and immune homeostasis [146, 147].

Furthermore, impairment of the immunological barrier diminishes microbial control mediated by the sIgA/pIgR system and the IL‐17/IL‐22 axis, as well as antimicrobial molecule induction, mucus maintenance, and epithelial repair, thereby promoting alterations in the microbiota, weakening of the mucus layer, and increased epithelial permeability [142, 148, 149]. Thus, the four intestinal barriers should be understood not as a hierarchical system, but as a highly interdependent network in which the failure of one barrier mechanism can trigger a cycle of dysfunction across the others.

## 3. Systemic Effects of the Intestinal Barrier Disruption

**Figure 3:**
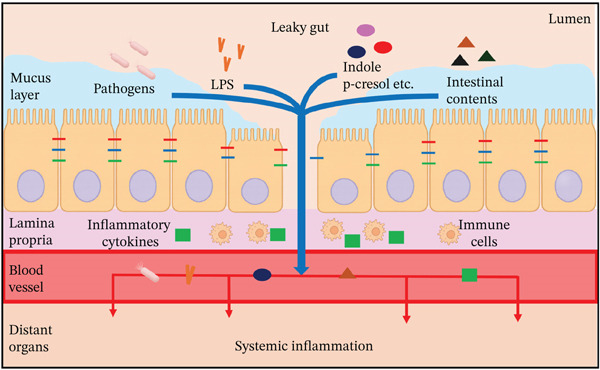
Leaky gut induces systemic inflammation. Disruption of the intestinal barrier allows luminal contents to enter the circulation and induce inflammation in distant tissues. Pathologically increased intestinal permeability (“leaky gut”) permits pathogens, lipopolysaccharide (LPS), uremic toxin precursors (such as indole and p‐cresol), and other intestinal contents to translocate into the lamina propria and bloodstream, from which they can reach distant tissues via the systemic circulation. In addition, the influx of luminal substances excessively activates immune cells in the lamina propria, and the inflammatory cytokines they produce likewise enter the circulation, reach distant tissues, and promote inflammation.

Disruption of the intestinal barrier by various factors increases intestinal permeability. For instance, the consumption of processed foods or alcohol, psychological stress, and the use of nonsteroidal anti‐inflammatory drugs (NSAIDs) have all been associated with increased intestinal permeability [150–153]. This allows substances from the intestinal lumen to enter the bloodstream, leading not only to intestinal diseases but also to the development and aggravation of systemic conditions [154]. In addition, the increased intestinal permeability activates immune cells within the lamina propria, which induces mucosal inflammation that further exacerbates barrier dysfunction [155]. In turn, this inflammation promotes the migration of immune cells and the entry of inflammatory cytokines into the bloodstream, extending the immune response beyond the gut (Figure 3). The resulting increase in circulating inflammatory cytokines promotes systemic inflammation.

Recent research has highlighted the relationships between the intestinal barrier, gut microbiota, and distant organs such as the liver, brain, skeletal muscle, and kidneys, revealing the interplay between intestinal barrier disruption and the health of these tissues (Table 3). Moreover, accurately assessing intestinal barrier function is essential for clarifying its relationship with systemic tissues. However, a variety of methods and biomarkers are available for evaluating intestinal barrier function, each with distinct advantages, limitations, and target sites. This chapter provides an overview of the methods used to assess intestinal barrier function (Table 4) and describes the effects of intestinal barrier dysfunction on various organs.

**Table 3:** Organ‐specific systemic effects linked to intestinal barrier disruption.

Organ/axis	Animal models	Observed pathological changes	Proposed mechanisms	Evidence type	Directionality
Gut–liver axis	• Repeated DSS colitis in mice [156]	• Hepatic steatosis/fatty liver.	• Barrier disruption, portal LPS translocation, and hepatocyte TLR4/MyD88/IRAK/TRAF6/IKK/NF‐*κ*B signaling [157–160].	• Human: mainly association (MASLD severity vs. D‐lactate/endotoxin) [161, 162].	Predominantly unidirectional (gut to liver) in this section.
	• DSS − induced chronic colitis + resolvin D1 treatment [163]	• Dyslipidemia and impaired liver function.	• Increased IL‐1*β* and proapoptotic signaling (e.g., Bax).	• Animal: partial causality supported by DSS, ethanol–fungal product, and intervention studies [156, 163, 164].	
	• C57BL/6 mice, chronic ethanol feeding for 8 weeks ± antifungal treatment [164]	• Hepatic inflammation, apoptosis, and Kupffer cell activation.	• Fungal *β*‐glucan translocation → CLEC7A/Dectin‐1 on Kupffer cells, IL‐1*β*, and hepatocyte injury/steatosis [164].		

Gut–brain axis	• Male C57BL/6 mice, 2% DSS for 5 days [165]	• Brain inflammation and cognitive decline.	• Intestinal/systemic immune activation and inflammatory cytokines reaching the brain.	• Human: largely association/correlation [166–169].	Bidirectional conceptually, but evidence here is mainly gut to brain.
	• ASO Parkinson^′^s model mice + controls, cyclic 1% DSS for 52 days [170]	• Altered myelin‐related genes.	• Possible BBB impairment and neuroinflammation.	• Animal: Supportive but still limited; permeability was not directly measured in key DSS‐brain studies [171, 172].	
	• Female C57BL/6 mice, multicycle 3% DSS (Days 1–5, 8–12, 15–19, and 22–26) [171]	• Reduced hippocampal neurogenesis.	• TLR4/NF‐*κ*B activation in brain tissue has been implicated [165].	• Overall causality remains unresolved.	
	• DSS‐colitis mouse model with brain transcriptomic/myelination analyses [172]	• Low‐dose DSS did not worsen PD‐like brain pathology [170].	• Specific non‐LPS mediators remain unresolved.		

Gut–muscle axis	• Germ‐free versus SPF mice; microbiota or SCFA rescue [173]	• Reduced skeletal muscle mass/sarcopenia–like phenotype [174].	• Inflammatory cytokine‐driven proteolysis (MuRF1/MAFbx).	• Human: largely associative (frailty, grip strength, and barrier markers) [175, 176].	Mainly unidirectional (gut to muscle) in current barrier literature.
	• Male mice, 0.75% DSS for 14 days [174]	• Increased muscle inflammatory cytokines and MuRF1/MAFbx [174].	• Proposed effects of altered amino acid absorption on mTOR‐related pathways [177–182].	• Animal: mixed/inconsistent findings; no consensus [174, 183].	
	• Eight‐week‐old male C57BL/6J mice, 0%–1% DSS in drinking water under a nonfiber diet [183]	• In another DSS model, no clear change in muscle weight or MuRF1/MAFbx [183].	• SCFAs and bile acid–FXR signaling are protective [173, 184].	• Causal mediators remain unclear.	

Gut–kidney axis	• Netrin‐1 transgenic, WT, IL‐6 KO, and C57BL/6J mice; 3.5% DSS for 5 days [185]	• Increased intestinal permeability with elevated serum creatinine [185].	• Barrier disruption, increased systemic absorption of indole/p‐cresol, hepatic conversion to indoxyl sulfate/p‐cresyl sulfate, and renal toxicity [186–190].	• Human/clinical rationale exists for uremic toxins and CKD progression [186–190].	Bidirectional (gut and kidney).
	• Seven‐week‐old male BALB/c mice; 5% DSS for 7 ± 4 − day recovery [191]	• Albuminuria and increased renal Lcn2/kidney inflammatory markers [191].	• Possible contribution of LPS, although direct measurements are limited [185, 191].	• Animal studies support both gut to kidney and kidney to gut links [185, 191–194].	
	• Six‐week‐old ICR mice; intraperitoneal adenine 25–75 mg/kg for 21 days [194]	• CKD model shows reduced kidney function plus increased colonic permeability [193, 194].	• Reverse direction; CKD, luminal urea accumulation, urease‐derived ammonia/ammonium hydroxide, and barrier damage [188, 192–194].	• Mechanistic causality is stronger than for brain or muscle, but some mediators remain undefined.	

**Table 4:** Representative methods for assessing intestinal barrier dysfunction.

Method	Main readout	Advantages	Limitations/caveats
Lactulose–mannitol (L/M) or other sugar permeability tests	Mainly intestinal permeability, especially small‐intestinal permeability; the L/M ratio is commonly used as a functional readout.	Noninvasive; widely used in human studies; relatively well established.	Protocols are not fully standardized; results are affected by dose, fasting status, urine collection time, renal function, gastric emptying, and intestinal transit. The test does not directly assess permeability to large macromolecules.
FITC‐dextran (animal studies)	In vivo intestinal permeability after oral administration.	Simple, sensitive, and widely used in preclinical models.	Strongly influenced by molecular weight, dose, timing of blood sampling, body composition, and transit time; limited direct translational value.
Serum LPS/LBP/sCD14	Mainly microbial translocation and/or the host response to endotoxin exposure.	Blood‐based; useful for linking barrier dysfunction with systemic endotoxemia or inflammation.	Not direct measures of epithelial permeability; LPS is technically difficult to measure, whereas LBP and sCD14 are also influenced by systemic inflammatory status.
Fecal calprotectin	Mainly intestinal mucosal inflammation, especially neutrophil‐driven inflammation.	Noninvasive; clinically established; useful in IBD monitoring.	Not a direct permeability marker; affected by assay platform, sample handling, and cutoff definition.
D‐lactate	Supportive marker of microbial metabolism and possible barrier injury/translocation.	Blood‐based; may be useful in selected settings such as short bowel syndrome or intestinal ischemia.	Low specificity; influenced by dysbiosis, short bowel syndrome, and metabolic context.
Zonulin	Putative barrier‐related/tight junction–associated marker.	Convenient serum‐ or stool‐based assay; widely used in research.	Major controversy regarding assay validity; commercial ELISAs may not reliably detect true zonulin, and correlation with established permeability tests is inconsistent.
Imaging and histologic correlates (e.g., CLE/pCLE, tight junction proteins, mucus/goblet cell assessment)	Morphological and partly functional correlates of barrier dysfunction.	Provide site‐specific and mechanistic information; CLE offers real‐time in vivo assessment.	Invasive; interpretation may be operator‐dependent; morphologic abnormalities do not always correspond directly to functional permeability.

### 3.1. Methods for Assessing Intestinal Barrier Function

Comprehensively assessing intestinal barrier dysfunction using a single indicator is challenging; therefore, multiple assessment methods should be interpreted in a complementary manner. Available approaches include those that primarily evaluate intestinal permeability, such as the lactulose–mannitol test and FITC‐dextran administration in animal studies; those that reflect microbial translocation or the host response, such as serum LPS/LBP/sCD14 and D‐lactate; those that indicate mucosal inflammation, such as fecal calprotectin; and those that capture morphological or histological alterations, including confocal laser endomicroscopy (CLE)/prove‐based CLE and the assessment of TJ proteins, the mucus layer, and goblet cells (Table 4) [195–199].

However, each marker reflects a distinct biological aspect. For example, although sugar permeability tests are useful for assessing functional permeability, their protocols remain insufficiently standardized, whereas LPS/LBP/sCD14 may indicate microbial translocation or systemic inflammation but are not direct markers of epithelial permeability [195]. In addition, although fecal calprotectin is useful for evaluating intestinal inflammation, it does not directly measure permeability, and the validity of zonulin assays remains controversial [200]. Imaging‐ and histology‐based assessments provide site‐specific and mechanistic information, but their use is limited by invasiveness and by challenges in standardizing image interpretation and data analysis [196]. Therefore, the evaluation of intestinal barrier function should ideally integrate permeability assays, blood‐ and stool‐based biomarkers, and histological correlations. These measures are not interchangeable but are rather complementary indicators reflecting distinct aspects of intestinal barrier dysfunction.

Furthermore, although DSS is a widely utilized and well‐established tool for inducing intestinal barrier disruption, it is important to consider its specific experimental characteristics. The DSS‐treated mouse model remains a valuable experimental system for analyzing barrier disruption and subsequent inflammatory responses, as it effectively reproduces key clinical features, such as epithelial injury, mucosal damage, immune cell infiltration, diarrhea, and hematochezia [201].

However, researchers should be mindful that the initiating event in this model is primarily acute chemical attack to the epithelium, which differs from the complex immune system–mediated initiation typically seen in human IBD [201]. Additionally, because disease phenotype can be influenced by factors such as the molecular weight of DSS, mouse strain, and gut microbiota composition, standardizing these variables is essential for consistent results [202].

Furthermore, although acute DSS models provide a robust platform for studying innate immune‐driven acute inflammation and increased permeability, their rapid onset and severity may differ from the more gradual, chronic time‐course of most human inflammatory conditions [203]. Although repeated DSS exposure models have been suggested to better reflect the immunological features and relapse‐remission cycles of human ulcerative colitis, they represent a simplified model of complex human pathophysiology [204]. Accordingly, findings from DSS models are most informative when interpreted alongside other experimental models and human clinical data, ensuring a comprehensive understanding of the intestinal barrier in translational research.

### 3.2. Liver (Gut–Liver Axis)

The liver, the largest organ in the human body, performs several vital functions, including the synthesis of plasma proteins (e.g., albumin), nutrient storage, detoxification of harmful substances, and bile production [205]. The gut–liver axis, first proposed in 1987, has been extensively investigated, with related interconnections being widely reported since the early 2000s [206, 207]. Many studies have focused on its association with MASLD, as intestinal barrier disruption is a contributing factor to its pathogenesis [208].

MASLD (previously known as NAFLD) is characterized by hepatic steatosis in individuals who have metabolic risk factors, consume little or no alcohol, and lack other causes of steatosis or liver disease [209]. As the disease advances, it can progress to metabolic dysfunction–associated steatohepatitis (MASH) (previously known as NASH) and subsequently to more severe conditions such as cirrhosis or liver cancer [210]. In humans, the severity of MASLD correlates with intestinal permeability. Specifically, D‐lactic acid, a marker of intestinal permeability, shows a positive correlation with markers of liver injury, including aspartate aminotransferase (AST), alanine transaminase (ALT), *γ*‐glutamyltransferase (*γ*‐GTP), and bilirubin [161]. Furthermore, meta‐analytic evidence indicates that circulating endotoxin levels, an indicator of intestinal permeability, are elevated in individuals with MASLD compared with healthy controls [162].

In animal studies, repeated administration of DSS, a compound that is widely used to induce colitis and intestinal barrier disruption in mice, not only exacerbates colitis and intestinal permeability in a concentration‐dependent manner, but also leads to impaired liver function and the development of hepatic steatosis (fatty liver) [156]. The administration of resolvin D1, a metabolite of omega‐3 polyunsaturated fatty acids, to mice with DSS‐induced colitis has been shown to improve intestinal barrier function, ameliorate colitis, and suppress the development of fatty liver [163].

DSS‐induced disruption of the intestinal barrier allows luminal LPS to enter the bloodstream [157]. Once LPS reaches the liver via the portal circulation, it activates toll‐like Receptor 4 (TLR4), which is expressed on hepatocytes. This activation triggers a downstream signaling cascade involving the adaptor protein myeloid differentiation Factor 88 (MyD88). MyD88 recruits IL‐1 receptor‐associated kinases (IRAKs), and phosphorylated IRAKs associate with tumor necrosis factor receptor‐associated Factor 6 (TRAF6). TRAF6 then activates nuclear factor‐kappa B (NF‐*κ*B) through I*κ*B‐kinase (IKK) complex‐mediated phosphorylation and degradation of I‐kappa‐B (I*κ*B), an inhibitor of NF‐*κ*B. Activated NF‐*κ*B translocates to the nucleus, where it promotes the expression of inflammatory cytokines, such as IL‐1*β*, and apoptosis‐related proteins, such as Bax, thereby enhancing hepatic inflammation and apoptosis [158, 159]. The MyD88‐independent downstream pathway of TLR4 is also known to activate transforming growth factor beta‐activated kinase 1 (TAK1) to promote the transcription of interferon genes [160].

Although LPS is the most frequently reported factor contributing to liver dysfunction associated with increased intestinal permeability, substances other than LPS have also been implicated. In mice, chronic alcohol administration increases the intestinal fungal burden, allowing *β*‐glucan, a component of the fungal cell wall, to translocate into the systemic circulation, which induces liver dysfunction. *β*‐glucan stimulates IL‐1*β* production via CLEC7A, a membrane receptor on hepatic Kupffer cells that recognizes fungal cell wall components, thereby promoting liver injury and hepatic steatosis. Administration of antifungal agents suppresses abnormal intestinal fungal overgrowth and the translocation of *β*‐glucan into the bloodstream, thereby ameliorating liver dysfunction [164].

High‐fat diet (HFD) feeding has traditionally been a common method for studying the effects of intestinal barrier disruption on the liver [211]. However, this model can directly affect the metabolic pathways of dietary fats in the liver, making it difficult to isolate the primary consequences of intestinal barrier dysfunction itself. In contrast, models that directly induce intestinal barrier disruption, such as DSS administration, allow for a more precise evaluation of its specific effects on the liver. To date, many studies have primarily focused on LPS‐mediated mechanisms, whereas the roles of other substances translocated from the intestine to the liver remain poorly understood. Future investigations into the effects of non‐LPS molecules on the liver following intestinal barrier disruption are expected to uncover new therapeutic targets for preventing the decline of liver function.

### 3.3. Brain (Gut–Brain Axis)

The gut–brain axis facilitates bidirectional communication through bottom‐up signaling (gut to brain) and top‐down signaling (brain to gut) [212]. For instance, IBS is strongly influenced by stress. In IBS, stress signals originating from the brain can trigger abnormal bowel movements, whereas visceral pain signals are transmitted from the gut back to the brain [213].

It has been reported that the gut microbiota is associated with the pathogenesis and exacerbation of psychiatric and neurodegenerative disorders, including autism, schizophrenia, and Parkinson′s disease [214]. Although the link between the intestinal barrier and the brain is less established than that of the gut microbiota–brain axis, it has become an area of increasing research interest. For instance, increased intestinal permeability has been observed in patients with psychiatric conditions, such as mood disorders, schizophrenia, and anxiety disorders [166]. Increased intestinal permeability can lead to excessive intestinal inflammation. This allows inflammatory cytokines to enter the circulation and reach the blood–brain barrier (BBB), where they can induce neuroinflammation and disrupt BBB integrity [167, 168]. A compromised BBB may permit the entry of various substances into the brain, potentially causing chronic neuroinflammation and a decline in cognitive function [169]. However, the causal relationship and detailed mechanisms remain unclear; it is not yet fully understood whether increased intestinal permeability is a cause or a consequence of psychiatric disorders.

Alcohol consumption has been reported to induce intestinal barrier disruption and alter the gut microbiota. This allows microbial products, such as LPS, to translocate into the bloodstream, subsequently inducing neuroinflammation and impairing brain function [215]. Furthermore, DSS‐induced intestinal permeability in mice has been associated with cognitive impairments measured by the Y‐maze and Morris water maze tests [165], suggesting that cognitive impairment may result from intestinal inflammation. In addition, DSS administration increases the expression of inflammation‐related proteins (e.g., TLR4 and phosphorylated NF‐*κ*B) and apoptosis‐related proteins in the brain, as well as the colon. It is known that the activation of the TLR4/NF‐*κ*B signaling pathway in the brain can trigger neuroinflammation, leading to a decline in cognitive function. In contrast, chronic administration of low concentration of DSS (1% DSS for 7 days, followed by 14 days of water, repeated for 3 cycles) in both wild‐type and Parkinson′s disease model mice (ASO mice) reportedly had no significant effect on Parkinson′s disease indicators, such as microglial cell number [170]. Thus, low concentration DSS increases intestinal permeability and provokes mild mucosal inflammation without causing systemic inflammation, but this is likely insufficient to exacerbate neurodegenerative conditions like Parkinson′s disease.

In addition, DSS‐induced colitis in mice activates both the intestinal and systemic immune systems, leading to brain inflammation accompanied by declines in cognitive function and altered expression of myelin‐related genes. These impairments in brain function have been attributed to inflammatory cytokines rather than LPS, suggesting that factors other than LPS may be involved [171, 172]. However, because intestinal permeability was not assessed in these studies, it remains difficult to conclude definitively that these effects were caused solely by the disruption of the intestinal barrier.

Elucidating the relationship between brain function and the intestinal barrier is challenging due to their bidirectional interactions, which make it difficult to distinguish primary effects from consequences and to establish causality. Nevertheless, given the evidence that intestinal barrier disruption can contribute to cognitive decline, maintaining barrier integrity may be a viable strategy for reducing the prevalence of psychiatric and neurological disorders. However, most of these findings are based on correlations, and neither the identification of specific factors that adversely affect the brain nor the clarification of causal relationships has been fully achieved. In addition, because cognitive decline is likely to progress with age, it will be necessary to develop strategies to maintain intestinal barrier function despite age‐related deterioration. As our society ages, it will become increasingly necessary to explore strategies to prevent cognitive decline. A more detailed understanding of the mechanisms linking the intestinal barrier and the brain is expected to reveal novel therapeutic agents capable of preserving brain function by improving intestinal barrier integrity.

### 3.4. Muscle (Gut–Muscle Axis)

Skeletal muscle is essential for physical activity and serves other important physiological roles, such as acting as a protein reservoir and contributing to thermogenesis. Furthermore, recent studies have shown that muscle tissue secretes biologically active substances known as myokines, which exert systemic effects [216].

The relationship between muscle and the gut microbiota is highlighted by the observation that GF mice, which lack gut bacteria, exhibit reduced muscle mass compared with SPF mice. This suggests that the gut microbiota plays a role in maintaining muscle mass [173]. This effect is likely mediated by SCFAs, as muscle mass in GF mice can be restored by transplanting gut microbiota from SPF mice or by orally administering SCFAs. Bile acids metabolized by the gut microbiota are also known to maintain muscle mass and strength via farnesoid X receptor (FXR) signaling [184].

Although less studied than the gut microbiota–muscle axis, the relationship between the intestinal barrier and muscle has gradually been attracting attention in recent years. Frailty is defined as a state of increased vulnerability to stressors resulting from aging‐associated functional declines and is often accompanied by declines in muscle function, such as reduced strength and slowed motor performance [217, 218]. A clinical study comparing healthy and frail elderly individuals found that frail individuals had higher circulating levels of zonulin, a marker of intestinal permeability, as well as inflammatory cytokines such as TNF‐*α* and IL‐6 [175]. Frailty in older adults is often associated with reduced gut microbial diversity and a lower abundance of SCFA‐producing bacteria. Furthermore, a study in middle‐aged and older adults shows a negative correlation between grip strength and diamine oxidase, another marker of intestinal permeability, in men [176]. This suggests that increased intestinal permeability may contribute to an age‐dependent decline in muscle strength.

In animal studies, the administration of a low concentration (0.75%) of DSS in drinking water for 14 days induces a sarcopenia‐like condition, characterized by a decrease in skeletal muscle mass [174]. This study demonstrates that DSS‐induced colitis increases the levels of inflammatory cytokines in skeletal muscle and upregulates the expression of muscle RING finger protein‐1 (MuRF1) and muscle atrophy F‐box (MAFbx), E3 ubiquitin ligases that promote muscle atrophy by proteasomal degradation of the target proteins [219]. In contrast, another study indicates that repeated administration of 1.0% DSS decreases the gene expression of intestinal TJ proteins but shows no significant changes in skeletal muscle weight or the expression of MuRF1 and MAFbx [183]. Thus, there is currently no consensus regarding the relationship between the intestinal barrier and skeletal muscle.

When the intestinal barrier is disrupted, the capacity of intestinal epithelial cells to absorb amino acids may be altered [177, 178]. Mechanistic target of rapamycin (mTOR), a key regulator of transcription, cell growth, proliferation, and repair, is activated by amino acids and is known to influence insulin secretion from pancreatic *β* cells, thereby affecting processes such as glucose uptake in muscle [179–181]. Furthermore, mTOR activation in skeletal muscle during aging or chronic disease has been associated with reduced muscle quality. Accordingly, alterations in the composition and quantity of amino acids entering the body due to intestinal barrier disruption may contribute to declines in muscle strength and mass via mTOR‐related pathways [182]. Thus, intestinal barrier disruption may also indirectly exert adverse effects on skeletal muscle through changes in the absorption of luminal substances.

The relationship between intestinal permeability and skeletal muscle remains poorly understood. Moreover, most of the currently available evidence is based on correlations, and in many cases the causal roles of specific bacteria or metabolites remain unclear. Therefore, future studies are needed to clarify why increased intestinal permeability leads to a reduction in skeletal muscle mass and to identify the causative factors responsible for this process. Elucidating the causal relationship underlying the gut–muscle axis may enable the development of interventions to mitigate frailty and, as a consequence, improve the quality of life in aging populations.

### 3.5. Kidney (Gut–Kidney Axis)

The kidneys are important organs that maintain homeostasis by filtering the blood to regulate the body′s fluid volume and composition while excreting metabolic waste products [220]. Among the substances cleared by the kidneys are uremic toxins, which include metabolites produced by the gut microbiota and subsequently modified by the liver [186]. Notably, indoxyl sulfate and p‐cresyl sulfate, which are derived from the microbial metabolites, indole and p‐cresol, respectively, can exert various toxic effects when they accumulate in the circulation [187]. A healthy intestinal barrier limits the systemic absorption of indole and p‐cresol, but when the barrier is disrupted, larger quantities of these microbial metabolites can enter the bloodstream, leading to increased production of uremic toxins [188].

This increase in uremic toxins is implicated in the development and progression of kidney diseases, such as chronic kidney disease (CKD) [189]. Furthermore, indoxyl sulfate–mediated impairment of kidney can elevate the concentration of uremic toxins in the blood, creating a vicious cycle [190]. In animal studies, exposure to 3.5% DSS for 5 days increases intestinal permeability and concomitantly elevates serum creatinine, a marker of renal function [185]. Additionally, 7 days administration of 5.0% DSS elevates fecal albumin, an indicator of intestinal permeability, and increases renal expression of lipocalin‐2 (Lcn2), a biomarker of kidney injury [191]. However, few studies have quantified circulating LPS or indoxyl sulfate, leaving the mechanism of the resulting renal injury unresolved.

Impaired kidney function is also known to cause intestinal barrier disruption. As the excretory function of the kidney declines, urea accumulates in the blood and is subsequently secreted into the intestinal lumen [192]. In the lumen, urea is metabolized into ammonia by urease‐producing gut bacteria and is subsequently converted to ammonium hydroxide. Both ammonia and ammonium hydroxide are cytotoxic and can disrupt the intestinal barrier, thereby increasing its permeability [188]. For instance, in a CKD model induced by intraperitoneal administration of adenine for 21 days, mice exhibit both decreased kidney function and increased colonic permeability [193, 194]. In this model, the increased permeability is attributed to the luminal accumulation of urea and its subsequent conversion to ammonia and ammonium hydroxide, which damages the intestinal barrier.

Thus, the kidneys and the intestinal barrier engage in a bidirectional cross talk, mediated in part by the gut microbiota, where dysfunction in one organ can promote dysfunction in the other. It has been established that disruption of the intestinal barrier increases circulating levels of uremic toxins and that administration of these uremic toxins to mice results in impaired renal function. However, the comprehensive mechanisms and causal pathways by which intestinal barrier disruption leads to kidney injury remain to be fully elucidated. Future challenges include identifying factors other than uremic toxins that contribute to renal dysfunction following intestinal barrier disruption, as well as elucidating the molecular mechanisms underlying this process. Given that an estimated 9.1% of the global population was affected by CKD in 2017 [221], strategies aimed at maintaining the intestinal barrier are considered important for preventing the progression of this disease.

## 4. Therapeutic Strategies for the Intestinal Barrier

Interventions targeting the intestinal barrier often affect multiple barrier layers simultaneously, rather than selectively targeting a single component of the physical, chemical, microbiological, or immunological barriers [222]. At present, many studies use barrier‐related surrogate markers, such as LPS, zonulin, TEER, and intestinal fatty acid‐binding protein (I‐FABP), as primary endpoints; however, studies evaluating barrier‐restoring therapies based on clinical hard outcomes remain limited [197]. This section provides an overview of interventions targeting the intestinal barrier, including dietary approaches, prebiotics, probiotics, synbiotics, postbiotics, bile acid modulators, anti‐inflammatory and immunomodulatory strategies, and lifestyle factors (Table 5).

**Table 5:** Therapeutic interventions targeting intestinal barrier dysfunction.

Category	Intervention	Targeted barrier	Evidence type	Risks/limitations
Dietary interventions	Increased dietary fiber, restriction of emulsifiers, and reduced alcohol intake	Mainly microbiological and chemical barriers; secondarily physical and immunological barriers	Some studies suggest benefit, but overall human evidence remains limited.	Strongly influenced by background diet, duration, and adherence.
Prebiotics/probiotics/synbiotics	Prebiotics, probiotics, and synbiotics	Mainly the microbiological barrier; secondarily chemical, immunological, and physical barriers	Meta‐analyses support reductions in LPS and zonulin.	Substantial heterogeneity in strains, doses, and study populations.
Postbiotics	SCFAs, inactivated microbial products, and microbial metabolites	Mainly physical, chemical, and immunological barriers; secondarily the microbiological barrier	Strong mechanistic rationale, but clinical studies remain early stage.	Product definition, standardization, and efficacy assessment remain unresolved.
Bile acid modulators	FXR/TGR5 modulation, including FXR agonists	Potentially all barrier layers	Promising in reviews and preclinical studies, but direct human evidence is limited.	Few studies assess barrier function as a primary endpoint.
Anti‐inflammatory/immunomodulatory therapies	Anti‐TNF, anti‐IL‐12/23, anti‐IL‐23, integrin blockade, and JAK inhibitors	Mainly the immunological barrier; secondarily, other barrier layers	Strong RCT evidence for disease control, whereas barrier improvement is usually secondary.	Not direct barrier‐repair agents.
Lifestyle factors	Stress reduction and sleep correction	Potentially all four barrier layers	Supported by preclinical studies, but human findings are inconsistent.	Difficult to standardize and highly susceptible to confounding factors.

### 4.1. Dietary Interventions (Including Restrictions)

Dietary intervention is one of the most fundamental strategies for restoring the intestinal barrier. Representative approaches include increasing the intake of fermentable dietary fiber, restricting ultraprocessed foods rich in emulsifiers, and reducing alcohol consumption. These interventions primarily target microbiological and chemical barriers, but they may also affect the physical and immunological barriers through enhanced SCFA production, reinforcement of the mucus layer, increased expression of TJ proteins, and suppression of inflammation [223].

A 2024 study in healthy individuals reported that although robust human evidence consistently demonstrating the efficacy of dietary interventions as a whole remains limited, some specific interventions, such as inulin, tend to show beneficial effects on intestinal barrier. In addition, an exploratory placebo‐controlled randomized controlled trial (RCT) conducted in 2025 found that 4 weeks of supplementation with carrageenan, a common emulsifier, reduced SCFA levels and significantly increased intestinal permeability from baseline [224].

With regard to alcohol, recent reviews and preclinical studies support its role in disrupting the intestinal barrier through direct damage to the physical and chemical barriers, as well as through the induction of dysbiosis. However, the effects of background diet, food matrix, intervention duration, and adherence are substantial, and thus conclusions remain inconsistent, particularly in studies involving healthy individuals [225]. Therefore, although dietary interventions are promising, it remains challenging to position them as a broadly applicable strategy at present.

### 4.2. Prebiotics, Probiotics, and Synbiotics

Prebiotics, probiotics, and synbiotics primarily target the microbiological barrier, whereas secondarily reinforcing the chemical, immunological, and physical barriers through the production of SCFAs, AMPs, mucin, and IgA, as well as the regulation of TJs. A 2025 systematic review and meta‐analysis showed that probiotics and synbiotics significantly reduced LPS levels across 24 trials and zonulin levels in 13 trials. Prebiotics were likewise associated with reductions in LPS in 16 RCTs, making this one of the most extensively studied groups in the clinical literature based on surrogate markers [226].

On the other hand, substantial heterogeneity in bacteria strains, dosages, durations of administration, substrates, target diseases, and evaluation markers remains a major limitation that hinders generalization. Furthermore, safety assessment is also important for probiotic preparations, and a 2024 risk review identified opportunistic infections and immunological adverse events as key concerns [227].

### 4.3. Postbiotics (Including SCFAs)

Postbiotics comprise preparations containing SCFAs such as butyric acid, acetic acid, and propionic acid, as well as inactivated bacterial cells and microbe‐derived metabolites, and these have attracted attention because they act simultaneously on the physical, chemical, and immunological barriers. In particular, SCFAs contribute to barrier homeostasis through effects on intestinal epithelial cell metabolism, TJs, mucin, AMPs, anti‐inflammatory immune responses, and the IL‐22/Treg axis, thereby also promoting restoration of the microbiological barrier. A 2025 article in *Nature Reviews Microbiology* identified SCFAs as major products of dietary fiber fermentation and comprehensively summarized their local and immunomodulatory effects [228].

Furthermore, in a phase 2 RCT conducted in 2025 in patients with advanced alcohol‐related liver disease, the postbiotic formulation, ReFerm, showed signals of barrier improvement, including reductions in I‐FABP and improvements in fibrosis markers; however, the primary histological endpoint itself did not reach statistical significance [229]. Therefore, although postbiotics are considered to have an advantage over live biotherapeutics (probiotics) in terms of safety, their formulation definitions, component standardization, optimal routes of administration, and efficacy across different diseases remain insufficiently established. At present, they may be regarded as having strong mechanistic plausibility but are still in the early stages of clinical implementation.

### 4.4. Bile Acid Modulators

Bile acid modulators, which target bile acid signaling through FXR and TGR5, theoretically exert broad barrier‐modulating effects because they can influence all layers of the physical, chemical, microbiological, and immunological barriers. Recent studies have indicated that the bile acid–FXR/TGR5 axis is important for maintaining barrier integrity and that FXR agonists may represent a promising strategy for gastrointestinal diseases [230]. Preclinical studies have shown that obeticholic acid (OCA) improves TJs, reduces inflammation, and modulates the microbiota in rat models of short bowel syndrome, thereby alleviating intestinal barrier disruption [231].

However, human trials using the intestinal barrier function as a primary endpoint remain scarce, and current clinical experience is largely limited to hepatobiliary and metabolic indications. Thus, although bile acid modulators represent a promising translational target, they should currently be regarded as emerging rather than established therapeutic agents for intestinal barrier repair.

### 4.5. Anti‐Inflammatory and Immunomodulatory Therapies

Anti‐inflammatory and immunomodulatory therapies include anti‐TNF agents, anti‐IL‐12/23 agents, anti‐IL‐23 agents, integrin blockade, and JAK inhibitors. These agents primarily target the immunological barrier and may secondarily improve TJs, epithelial metabolism, mucus secretion, and microbial composition by suppressing inflammation. Although these drug classes have the strongest RCT evidence for disease control, barrier restoration often remains only a secondary endpoint [232]. In fact, a prospective human biopsy study published in 2024 showed that colonic TEER increased significantly after treatment with vedolizumab, a monoclonal antibody for *α*4*β*7‐integrin, and that this change correlated with clinical, endoscopic, and histologic improvement [233]. From this perspective, these interventions are more appropriately understood not as direct barrier‐repair drugs, but rather as strategies that promote barrier restoration as a result of inflammation control. However, it should be noted that some immunomodulatory approaches, such as cytokine administration, are theoretically promising for barrier repair but have not yet demonstrated sufficient clinical benefit.

### 4.6. Lifestyle Factors (Stress and Sleep)

Lifestyle factors, including stress and sleep, may also affect all four layers of the barrier through the hypothalamic–pituitary–adrenal (HPA) axis, autonomic signaling, and alterations in mucin, TJs, and the microbiota [234]. In preclinical studies, the association between stress and intestinal permeability has been relatively consistent, although a 2023 review concluded that the relationship between psychosocial stress and permeability in healthy humans is not necessarily consistent [234]. Regarding sleep, a 2024 mouse study showed that sleep deprivation reduced goblet cell numbers, mucin levels, and TJ proteins, and that these changes were ameliorated by melatonin [235]. In contrast, a 2023 study in healthy young men found that although severe short‐term sleep restriction decreased microbial richness, it did not significantly alter intestinal permeability itself.

Increased intestinal permeability has been reported in humans under conditions of combined stressors [236]. Nevertheless, standardizing such interventions is difficult, and major confounding factors, including diet, exercise, and circadian rhythm, must be considered. Therefore, at present, lifestyle modification is more appropriately positioned as an adjunctive rather than a stand‐alone strategy.

## 5. Conclusion

The maintenance of intestinal barrier integrity is crucial for the prevention and treatment of not only intestinal disorders but also a range of systemic diseases. This review has categorized the intestinal barrier into four key components—physical, chemical, microbiological, and immunological—and highlighted the critical factors contributing to each barrier function. Proteins associated with TJs and mucin, the primary constituent of the mucus layer, are fundamental to preserving this barrier. Moreover, the identification of novel antimicrobial substances, such as Lypd8, suggests that continued research in this field will yield further significant discoveries. We have also discussed the systemic consequences of a compromised intestinal barrier in the context of the gut–liver, gut–brain, gut–muscle, and gut–kidney axes. These axes often involve bidirectional interactions, underscoring the importance of identifying key mediators, such as LPS, that influence distant organ function. In the context of the gut–kidney axis, numerous translocated substances with detrimental effects have been identified, suggesting that therapeutic strategies targeting these molecules represent a promising area for future research. In addition, this review emphasizes that intestinal barrier dysfunction should be understood not as impairment of an isolated structure, but as disruption of a highly integrated network in which the physical, chemical, microbiological, and immunological barriers mutually regulate one another. From a therapeutic perspective, currently available interventions—including dietary approaches, microbiota‐directed strategies such as prebiotics, probiotics, synbiotics and postbiotics, bile acid modulators, anti‐inflammatory and immunomodulatory therapies, and lifestyle modification—appear to influence multiple barrier layers simultaneously rather than selectively targeting a single component. However, although many of these strategies show mechanistic promise, most current evidence still relies on surrogate markers such as LPS, zonulin, TEER, and I‐FABP, whereas studies demonstrating clinically meaningful barrier restoration based on hard outcomes remain limited. Accordingly, future progress in this field will require not only a deeper mechanistic understanding of barrier regulation, but also the establishment of standardized assessment methods and translational frameworks capable of linking barrier‐related biomarkers to clinically relevant outcomes.

Significant questions remain regarding the precise mechanisms by which the gut microbiota contributes to the disruption or maintenance of the intestinal barrier. The causal relationship is often ambiguous, making it difficult to determine whether alterations in the microbiota are a cause or a consequence of barrier dysfunction. However, elucidating these mechanisms is essential for the identification of effective therapeutic targets. Recent advances in metagenomic and metabolomic analyses are rapidly enhancing our understanding of the gut environment. The application of these technologies to investigate intestinal barrier disruption and its systemic effects is expected to drive significant progress in this field. Ultimately, a more comprehensive understanding of how intestinal barrier dysfunction varies according to intestinal region, disease context, and stage of progression, together with rigorous evaluation of barrier‐restoring interventions, will be essential for translating intestinal barrier research into effective preventive and therapeutic strategies for both intestinal and systemic diseases.

## Author Contributions

J.W.: conceptualization, literature search, visualization, writing—original draft, and review and editing. K.K.: literature search and review and editing. T.K.: conceptualization, literature search, review and editing, and supervision.

## Funding

This study was supported by the Meiji Holdings Co., Ltd and Japan Society for the Promotion of Science (10.13039/501100001691) (JP25K02271).

## Disclosure

All authors approved the final manuscript.

## Conflicts of Interest

J.W. and K.K. are employed by Meiji Holding Co., Ltd., a company that is a food and pharmaceutical business.

## Data Availability

Data sharing is not applicable to this article as no datasets were generated or analyzed during the current study.

## References

[1] Rath E. and Haller D. , Intestinal Epithelial Cell Metabolism at the Interface of Microbial Dysbiosis and Tissue Injury, Mucosal Immunology. (2022) 15, no. 4, 595–604, 10.1038/s41385-022-00514-x, 35534699. 35534699 PMC9259489

[2] Fernandez-Cantos M. V. , Garcia-Morena D. , Iannone V. , El-Nezami H. , Kolehmainen M. , and Kuipers O. P. , Role Of Microbiota And Related Metabolites In Gastrointestinal Tract Barrier Function In NAFLD, Tissue Barriers. (2021) 9, no. 3, 1879719, 10.1080/21688370.2021.1879719, 34280073. 34280073 PMC8489918

[3] Turner J. R. , Intestinal Mucosal Barrier Function in Health and Disease, Nature Reviews Immunology. (2009) 9, no. 11, 799–809, 10.1038/nri2653. 19855405

[4] Belkaid Y. and Hand T. W. , Role of the Microbiota in Immunity and Inflammation, Cell. (2014) 157, no. 1, 121–141, 10.1016/j.cell.2014.03.011, 24679531. 24679531 PMC4056765

[5] Rogers A. P. , Mileto S. J. , and Lyras D. , Impact of Enteric Bacterial Infections at and Beyond the Epithelial Barrier, Nature Reviews Microbiology. (2023) 21, no. 4, 260–274, 10.1038/s41579-022-00794-x, 36175770. 36175770

[6] Obrenovich M. E. M. , Leaky Gut, Leaky Brain?, Microorganisms. (2018) 6, no. 4, 10.3390/microorganisms6040107, 30340384. PMC6313445 30340384

[7] Camilleri M. and Vella A. , What To Do About The Leaky Gut, Gut. (2022) 71, no. 2, 424–435, 10.1136/gutjnl-2021-325428, 34509978. 34509978 PMC9028931

[8] Lacy B. E. , Wise J. L. , and Cangemi D. J. , Leaky Gut Syndrome: Myths and Management, Journal of Gastroenterology and Hepatology. (2024) 20, no. 5, 264–272. PMC11345991 39193076

[9] Valitutti F. , Mennini M. , Monacelli G. , Fagiolari G. , Piccirillo M. , Di Nardo G. , and Di Cara G. , Intestinal Permeability, Food Antigens And The Microbiome: A Multifaceted Perspective, Front Allergy. (2024) 5, 1505834, 10.3389/falgy.2024.1505834, 39850945. 39850945 PMC11754301

[10] Camilleri M. , Leaky Gut: Mechanisms, Measurement and Clinical Implications in Humans, Gut. (2019) 68, no. 8, 1516–1526, 10.1136/gutjnl-2019-318427, 31076401. 31076401 PMC6790068

[11] Vanuytsel T. , Tack J. , and Farre R. , The Role of Intestinal Permeability in Gastrointestinal Disorders and Current Methods of Evaluation, Frontiers in Nutrition. (2021) 8, 717925, 10.3389/fnut.2021.717925, 34513903. 34513903 PMC8427160

[12] Shi M. , Zong X. , Hur J. , Birmann B. M. , Martinez-Maza O. , Epeldegui M. , Chan A. T. , Giovannucci E. L. , and Cao Y. , Circulating Markers Of Microbial Translocation And Host Response To Bacteria With Risk Of Colorectal Cancer: A Prospective, Nested Case-Control Study In Men, EBioMedicine. (2023) 91, 104566, 10.1016/j.ebiom.2023.104566, 37075493. 37075493 PMC10131057

[13] Damianos J. A. , Bledsoe A. , Camilleri M. , and Murray J. A. , Coeliac Disease and the Intestinal Barrier: Mechanisms of Disruption and Strategies for Restoration, Gut. (2026) 75, no. 4, 826–838, 10.1136/gutjnl-2025-335373, 40579122. 40579122

[14] Grover M. , Vanuytsel T. , and Chang L. , Intestinal Permeability in Disorders of Gut-Brain Interaction: From Bench to Bedside, Gastroenterology. (2025) 168, no. 3, 480–495, 10.1053/j.gastro.2024.08.033, 39236897. 39236897

[15] Dissayabutra T. , Chuaypen N. , Somnark P. , Boonkaew B. , Udomkarnjananun S. , Kittiskulnam P. , Charoenchittang P. , Prombutara P. , and Tangkijvanich P. , Characterization Of Gut Dysbiosis And Intestinal Barrier Dysfunction In Patients With Metabolic Dysfunction-Associated Steatotic Liver Disease And Chronic Kidney Disease: A Comparative Study, Scientific Reports. (2025) 15, no. 1, 10.1038/s41598-025-00237-6, 40319096. PMC12049563 40319096

[16] Neurath M. F. , Artis D. , and Becker C. , The Intestinal Barrier: A Pivotal Role in Health, Inflammation, and Cancer, Lancet Gastroenterology & Hepatology. (2025) 10, no. 6, 573–592, 10.1016/S2468-1253(24)00390-X, 40086468. 40086468

[17] Peterson L. W. and Artis D. , Intestinal Epithelial Cells: Regulators of Barrier Function and Immune Homeostasis, Nature Reviews Immunology. (2014) 14, no. 3, 141–153, 10.1038/nri3608. 24566914

[18] Beumer J. and Clevers H. , Cell Fate Specification and Differentiation in the Adult Mammalian Intestine, Nature Reviews Molecular Cell Biology. (2021) 22, no. 1, 39–53, 10.1038/s41580-020-0278-0, 32958874. 32958874

[19] Gribble F. M. and Reimann F. , Function and Mechanisms of Enteroendocrine Cells and Gut Hormones in Metabolism, Nature Reviews Endocrinology. (2019) 15, no. 4, 226–237, 10.1038/s41574-019-0168-8. 30760847

[20] Menard S. , Cerf-Bensussan N. , and Heyman M. , Multiple Facets of Intestinal Permeability and Epithelial Handling of Dietary Antigens, Mucosal Immunology. (2010) 3, no. 3, 247–259, 10.1038/mi.2010.5, 20404811. 20404811

[21] Suzuki T. , Regulation of the Intestinal Barrier by Nutrients: The Role of Tight Junctions, Animal Science Journal.(2020) 91, no. 1, 10.1111/asj.13357, e13357, 32219956. 32219956 PMC7187240

[22] Tsukita S. , Tanaka H. , and Tamura A. , The Claudins: From Tight Junctions to Biological Systems, Trends in Biochemical Sciences. (2019) 44, no. 2, 141–152, 10.1016/j.tibs.2018.09.008. 30665499

[23] Lu Z. , Ding L. , Lu Q. , and Chen Y. H. , Claudins In Intestines: Distribution And Functional Significance In Health And Diseases, Tissue Barriers.(2013) 1, no. 3, e24978, 10.4161/tisb.24978, 24478939. 24478939 PMC3879173

[24] Kozieł M. J. , Ziaja M. , and Piastowska-Ciesielska A. W. , Intestinal Barrier, Claudins and Mycotoxins, Toxins. (2021) 13, no. 11, 10.3390/toxins13110758, 34822542. PMC8622050 34822542

[25] González-González M. , Díaz-Zepeda C. , Eyzaguirre-Velásquez J. , González-Arancibia C. , Bravo J. A. , and Julio-Pieper M. , Investigating Gut Permeability in Animal Models of Disease, Frontiers in Physiology. (2019) 9, 10.3389/fphys.2018.01962, 30697168. PMC6341294 30697168

[26] Tanaka H. , Takechi M. , Kiyonari H. , Shioi G. , Tamura A. , and Tsukita S. , Intestinal Deletion of Claudin-7 Enhances Paracellular Organic Solute Flux and Initiates Colonic Inflammation in Mice, Gut. (2015) 64, no. 10, 1529–1538, 10.1136/gutjnl-2014-308419, 25691495. 25691495

[27] Raju P. , Shashikanth N. , Tsai P. Y. , Pongkorpsakol P. , Chanez-Paredes S. , Steinhagen P. R. , Kuo W. T. , Singh G. , Tsukita S. , and Turner J. R. , Inactivation Of Paracellular Cation-Selective Claudin-2 Channels Attenuates Immune-Mediated Experimental Colitis In Mice, Journal of Clinical Investigation. (2020) 130, no. 10, 5197–5208, 10.1172/JCI138697, 32516134. 32516134 PMC7524482

[28] Furuse M. , Hirase T. , Itoh M. , Nagafuchi A. , Yonemura S. , Tsukita S. , and Tsukita S. , Occludin: A Novel Integral Membrane Protein Localizing At Tight Junctions, Journal Of Cell Biology. (1993) 123, no. 6, 1777–1788, 10.1083/jcb.123.6.1777, 8276896. 8276896 PMC2290891

[29] McCarthy K. M. , Skare I. B. , Stankewich M. C. , Furuse M. , Tsukita S. , Rogers R. A. , Lynch R. D. , and Schneeberger E. E. , Occludin Is a Functional Component of the Tight Junction, Journal of Cell Science. (1996) 109, no. 9, 2287–2298, 10.1242/jcs.109.9.2287. 8886979

[30] Saitou M. , Fujimoto K. , Doi Y. , Itoh M. , Fujimoto T. , Furuse M. , Takano H. , Noda T. , and Tsukita S. , Occludin-Deficient Embryonic Stem Cells Can Differentiate Into Polarized Epithelial Cells Bearing Tight Junctions, Journal of Cell Biology. (1998) 141, no. 2, 397–408, 10.1083/jcb.141.2.397, 9548718. 9548718 PMC2148457

[31] Saitou M. , Furuse M. , Sasaki H. , Schulzke J. D. , Fromm M. , Takano H. , Noda T. , and Tsukita S. , Complex Phenotype Of Mice Lacking Occludin, A Component Of Tight Junction Strands, Molecular Biology. (2000) 11, no. 12, 4131–4142, 10.1091/mbc.11.12.4131, 11102513. PMC15062 11102513

[32] Rao R. , Occludin Phosphorylation In Regulation Of Epithelial Tight Junctions, Annals of the New York Academy of Sciences. (2009) 1165, no. 1, 62–68, 10.1111/j.1749-6632.2009.04054.x, 19538289. 19538289 PMC6202026

[33] Cummins P. M. , Occludin: One Protein, Many Forms, Molecular and Cellular Biology. (2012) 32, no. 2, 242–250, 10.1128/MCB.06029-11, 22083955. 22083955 PMC3255790

[34] Martìn-Padura I. , Lostaglio S. , Schneemann M. , Williams L. , Romano M. , Fruscella P. , Panzeri C. , Stoppacciaro A. , Ruco L. , Villa A. , Simmons D. , and Dejana E. , Junctional Adhesion Molecule, A Novel Member Of The Immunoglobulin Superfamily That Distributes At Intercellular Junctions And Modulates Monocyte Transmigration, Journal of Cell Biology. (1998) 142, no. 1, 117–127, 10.1083/jcb.142.1.117, 9660867. 9660867 PMC2133024

[35] Luissint A. C. , Nusrat A. , and Parkos C. A. , JAM-Related Proteins In Mucosal Homeostasis And Inflammation, Seminars in Immunopathology. (2014) 36, no. 2, 211–226, 10.1007/s00281-014-0421-0, 24667924. 24667924 PMC4084508

[36] Laukoetter M. G. , Nava P. , Lee W. Y. , Severson E. A. , Capaldo C. T. , Babbin B. A. , Williams I. R. , Koval M. , Peatman E. , Campbell J. A. , Dermody T. S. , Nusrat A. , and Parkos C. A. , JAM-A Regulates Permeability and Inflammation in the Intestine In Vivo, Journal of Experimental Medicine. (2007) 204, no. 13, 3067–3076, 10.1084/jem.20071416, 18039951. 18039951 PMC2150975

[37] Fan S. , Weight C. M. , Luissint A. C. , Hilgarth R. S. , Brazil J. C. , Ettel M. , Nusrat A. , and Parkos C. A. , Role Of JAM-A Tyrosine Phosphorylation In Epithelial Barrier Dysfunction During Intestinal Inflammation, Molecular Biology of the Cell. (2019) 30, no. 5, 566–578, 10.1091/mbc.E18-08-0531, 30625033. 30625033 PMC6589701

[38] Ikenouchi J. , Furuse M. , Furuse K. , Sasaki H. , Tsukita S. , and Tsukita S. , Tricellulin Constitutes A Novel Barrier At Tricellular Contacts Of Epithelial Cells, Journal of Cell Biology. (2005) 171, no. 6, 939–945, 10.1083/jcb.200510043, 16365161. 16365161 PMC2171318

[39] Cho Y. , Haraguchi D. , Shigetomi K. , Matsuzawa K. , Uchida S. , and Ikenouchi J. , Tricellulin Secures the Epithelial Barrier at Tricellular Junctions by Interacting With Actomyosin, Journal of Cell Biology. (2022) 221, no. 4, 10.1083/jcb.202009037, e202009037, 35148372. 35148372 PMC8847807

[40] Higashi T. , Tokuda S. , Kitajiri S. , Masuda S. , Nakamura H. , Oda Y. , and Furuse M. , Analysis Of The ′Angulin′ Proteins LSR, ILDR1 And ILDR2--Tricellulin Recruitment, Epithelial Barrier Function And Implication In Deafness Pathogenesis, Journal of Cell Science. (2013) 126, no. 4, 966–977, 10.1242/jcs.116442, 23239027. 23239027

[41] Ikenouchi J. , Sasaki H. , Tsukita S. , Furuse M. , and Tsukita S. , Loss Of Occludin Affects Tricellular Localization Of Tricellulin, Molecular Biology of the Cell. (2008) 19, no. 11, 4687–4693, 10.1091/mbc.e08-05-0530, 18768749. 18768749 PMC2575184

[42] Zihni C. , Mills C. , Matter K. , and Balda M. S. , Tight Junctions: From Simple Barriers to Multifunctional Molecular Gates, Nature Reviews Molecular Cell Biology. (2016) 17, no. 9, 564–580, 10.1038/nrm.2016.80, 27353478. 27353478

[43] Fanning A. S. and Anderson J. M. , Zonula Occludens-1 And -2 Are Cytosolic Scaffolds That Regulate The Assembly Of Cellular Junctions, Annals of the New York Academy of Sciences. (2009) 1165, no. 1, 113–120, 10.1111/j.1749-6632.2009.04440.x, 19538295. 19538295 PMC3759978

[44] Nomme J. , Antanasijevic A. , Caffrey M. , Van Itallie C. M. , Anderson J. M. , Fanning A. S. , and Lavie A. , Structural basis of a key factor regulating the affinity between the zonula occludens first PDZ domain and claudins, Journal of Biological Chemistry. (2015) 290, no. 27, 16595–16606, 10.1074/jbc.M115.646695, 26023235. 26023235 PMC4505413

[45] Otani T. , Nguyen T. P. , Tokuda S. , Sugihara K. , Sugawara T. , Furuse K. , Miura T. , Ebnet K. , and Furuse M. , Claudins And JAM-A Coordinately Regulate Tight Junction Formation And Epithelial Polarity, Journal of Cell Biology. (2019) 218, no. 10, 3372–3396, 10.1083/jcb.201812157, 31467165. 31467165 PMC6781433

[46] Kuo W. T. , Zuo L. , Odenwald M. A. , Madha S. , Singh G. , Gurniak C. B. , Abraham C. , and Turner J. R. , The Tight Junction Protein ZO-1 Is Dispensable for Barrier Function but Critical for Effective Mucosal Repair, Gastroenterology. (2021) 161, no. 6, 1924–1939, 10.1053/j.gastro.2021.08.047, 34478742. 34478742 PMC8605999

[47] Meng W. and Takeichi M. , Adherens Junction: Molecular Architecture and Regulation, Cold Spring Harbor Perspectives in Biology. (2009) 1, no. 6, a002899, 10.1101/cshperspect.a002899, 20457565. 20457565 PMC2882120

[48] Kawauchi T. , Cell Adhesion and Its Endocytic Regulation in Cell Migration During Neural Development and Cancer Metastasis, International Journal of Molecular Sciences. (2012) 13, no. 4, 4564–4590, 10.3390/ijms13044564, 22605996. 22605996 PMC3344232

[49] Groschwitz K. R. and Hogan S. P. , Intestinal Barrier Function: Molecular Regulation And Disease Pathogenesis, Journal of Allergy and Clinical Immunology. (2009) 124, no. 1, 3–20, 10.1016/j.jaci.2009.05.038, 19560575. 19560575 PMC4266989

[50] Campàs O. , Noordstra I. , and Yap A. S. , Adherens Junctions as Molecular Regulators of Emergent Tissue Mechanics, Nature Reviews Molecular Cell Biology. (2024) 25, no. 4, 252–269, 10.1038/s41580-023-00688-7, 38093099. 38093099

[51] Nishimura Y. V. and Kawauchi T. , The Duality of Cdk 5: A Master Regulator in Neurodevelopment and a Hijacked Oncogene in Cancer, Cells. (2025) 14, no. 23, 10.3390/cells14231876, 41369365. PMC12691403 41369365

[52] Jawhari A. , Farthing M. , and Pignatelli M. , The Importance Of The E-Cadherin-Catenin Complex In The Maintenance Of Intestinal Epithelial Homoeostasis: More Than Intercellular Glue?, Gut. (1997) 41, no. 5, 581–584, 10.1136/gut.41.5.581, 9414960. 9414960 PMC1891567

[53] Shapiro L. and Weis W. I. , Structure And Biochemistry Of Cadherins And Catenins, Cold Spring Harbor Perspectives in Biology. (2009) 1, no. 3, a003053, 10.1101/cshperspect.a003053, 20066110. 20066110 PMC2773639

[54] Bondow B. J. , Faber M. L. , Wojta K. J. , Walker E. M. , and Battle M. A. , E-Cadherin Is Required For Intestinal Morphogenesis In The Mouse, Developmental Biology. (2012) 371, no. 1, 1–12, 10.1016/j.ydbio.2012.06.005, 22766025. 22766025 PMC3455111

[55] Schneider M. R. , Dahlhoff M. , Horst D. , Hirschi B. , Trülzsch K. , Müller-Höcker J. , Vogelmann R. , Allgäuer M. , Gerhard M. , Steininger S. , Wolf E. , and Kolligs F. T. , A Key Role For E-Cadherin In Intestinal Homeostasis And Paneth Cell Maturation, PLoS One. (2010) 5, no. 12, e14325, 10.1371/journal.pone.0014325, 21179475. 21179475 PMC3001873

[56] Grill J. I. , Neumann J. , Hiltwein F. , Kolligs F. T. , and Schneider M. R. , Intestinal E-Cadherin Deficiency Aggravates Dextran Sodium Sulfate-Induced Colitis, Digestive Diseases and Sciences. (2015) 60, no. 4, 895–902, 10.1007/s10620-015-3551-x, 25634675. 25634675

[57] Takai Y. and Nakanishi H. , Nectin and Afadin: Novel Organizers of Intercellular Junctions, Journal of Cell Science. (2003) 116, no. 1, 17–27, 10.1242/jcs.00167, 12456712. 12456712

[58] Hoshino T. , Sakisaka T. , Baba T. , Yamada T. , Kimura T. , and Takai Y. , Regulation Of E-Cadherin Endocytosis By Nectin Through Afadin, Rap 1, And P120ctn, Journal of Biological Chemistry. (2005) 280, no. 25, 24095–24103, 10.1074/jbc.M414447200, 15857834. 15857834

[59] Irie K. , Shimizu K. , Sakisaka T. , Ikeda W. , and Takai Y. , Roles and Modes of Action of Nectins in Cell-Cell Adhesion, Seminars in Cell and Developmental Biology. (2004) 15, no. 6, 643–656, 10.1016/S1084-9521(04)00088-6, 15561584. 15561584

[60] Tanaka-Okamoto M. , Hori K. , Ishizaki H. , Itoh Y. , Onishi S. , Yonemura S. , Takai Y. , and Miyoshi J. , Involvement Of Afadin In Barrier Function And Homeostasis Of Mouse Intestinal Epithelia, Journal of Cell Science. (2011) 124, no. 13, 2231–2240, 10.1242/jcs.081000, 21652626. 21652626 PMC3115770

[61] Schlegel N. , Boerner K. , and Waschke J. , Targeting Desmosomal Adhesion And Signalling For Intestinal Barrier Stabilization In Inflammatory Bowel Diseases-Lessons From Experimental Models And Patients, Acta Physiologica. (2021) 231, no. 1, e13492, 10.1111/apha.13492, 32419327. 32419327

[62] Hegazy M. , Perl A. L. , Svoboda S. A. , and Green K. J. , Desmosomal Cadherins in Health and Disease, Annual Review of Pathology. (2022) 17, no. 1, 47–72, 10.1146/annurev-pathol-042320-092912, 34425055. PMC8792335 34425055

[63] Holthöfer B. , Windoffer R. , Troyanovsky S. , and Leube R. E. , Structure and Function of Desmosomes, International Review of Cytology. (2007) 264, 65–163, 10.1016/S0074-7696(07)64003-0. 17964922

[64] Bharathan N. K. , Mattheyses A. L. , and Kowalczyk A. P. , The Desmosome Comes Into Focus, Journal of Cell Biology. (2024) 223, no. 9, e202404120, 10.1083/jcb.202404120, 39120608. 39120608 PMC11317759

[65] Gross A. , Pack L. A. P. , Schacht G. M. , Kant S. , Ungewiss H. , Meir M. , Schlegel N. , Preisinger C. , Boor P. , Guldiken N. , Krusche C. A. , Sellge G. , Trautwein C. , Waschke J. , Heuser A. , Leube R. E. , and Strnad P. , Desmoglein 2, But Not Desmocollin 2, Protects Intestinal Epithelia From Injury, Mucosal Immunology. (2018) 11, no. 6, 1630–1639, 10.1038/s41385-018-0062-z. 30115995

[66] Flemming S. , Luissint A. C. , Kusters D. H. M. , Raya-Sandino A. , Fan S. , Zhou D. W. , Hasegawa M. , Garcia-Hernandez V. , García A. J. , Parkos C. A. , and Nusrat A. , Desmocollin-2 Promotes Intestinal Mucosal Repair By Controlling Integrin-Dependent Cell Adhesion And Migration, Molecular Biology of the Cell. (2020) 31, no. 6, 407–418, 10.1091/mbc.E19-12-0692, 31967937. 31967937 PMC7185897

[67] Kang Y. , Park H. , Choe B. H. , and Kang B. , The Role and Function of Mucins and Its Relationship to Inflammatory Bowel Disease, Frontiers in Medicine. (2022) 9, 848344, 10.3389/fmed.2022.848344, 35602503. 35602503 PMC9120656

[68] Corfield A. P. , Mucins: A Biologically Relevant Glycan Barrier In Mucosal Protection, Biochimica et Biophysica Acta. (2015) 1850, no. 1, 236–252, 10.1016/j.bbagen.2014.05.003, 24821013. 24821013

[69] Gum J. R. , Crawley S. C. , Hicks J. W. , Szymkowski D. E. , and Kim Y. S. , MUC17, a Novel Membrane-Tethered Mucin, Biochemical and Biophysical Research Communications. (2002) 291, no. 3, 466–475, 10.1006/bbrc.2002.6475, 11855812. 11855812

[70] Johansson M. E. , Sjövall H. , and Hansson G. C. , The Gastrointestinal Mucus System In Health And Disease, Nature Reviews Gastroenterology & Hepatology. (2013) 10, no. 6, 352–361, 10.1038/nrgastro.2013.35, 23478383. 23478383 PMC3758667

[71] Johansson M. E. , Phillipson M. , Petersson J. , Velcich A. , Holm L. , and Hansson G. C. , The Inner of the Two Muc 2 Mucin-Dependent Mucus Layers in Colon Is Devoid of Bacteria, Proceedings of the National Academy of Sciences. (2008) 105, no. 39, 15064–15069, 10.1073/pnas.0803124105, 18806221. PMC2567493 18806221

[72] Tytgat K. M. , Büller H. A. , Opdam F. J. , Kim Y. S. , Einerhand A. W. , and Dekker J. , Biosynthesis of Human Colonic Mucin: Muc2 Is the Prominent Secretory Mucin, Gastroenterology. (1994) 107, no. 5, 1352–1363, 10.1016/0016-5085(94)90537-1, 7926500. 7926500

[73] Johansson M. E. , Ambort D. , Pelaseyed T. , Schutte A. , Gustafsson J. K. , Ermund A. , Subramani D. B. , Holmén-Larsson J. M. , Thomsson K. A. , Bergström J. H. , van der Post S. , Rodriguez-Piñeiro A. M. , Sjövall H. , Bäckström M. , and Hansson G. C. , Composition and Functional Role of the Mucus Layers in the Intestine, Cellular and Molecular Life Sciences. (2011) 68, no. 22, 3635–3641, 10.1007/s00018-011-0822-3, 21947475. 21947475 PMC11114784

[74] Walsh M. D. , Clendenning M. , Williamson E. , Pearson S. A. , Walters R. J. , Nagler B. , Packenas D. , Win A. K. , Hopper J. L. , Jenkins M. A. , Haydon A. M. , Rosty C. , English D. R. , Giles G. G. , McGuckin M. A. , Young J. P. , and Buchanan D. D. , Expression of MUC2, MUC5AC, MUC5B, and MUC6 Mucins in Colorectal Cancers and Their Association With the CpG Island Methylator Phenotype, Modern Pathology. (2013) 26, no. 12, 1642–1656, 10.1038/modpathol.2013.101, 23807779. 23807779

[75] Sala-Rabanal M. , Yurtsever Z. , Nichols C. G. , and Brett T. J. , Secreted CLCA1 Modulates TMEM16A To Activate Ca(2+)-Dependent Chloride Currents In Human Cells, Elife. (2015) 4, e05875, 10.7554/eLife.05875, 25781344. 25781344 PMC4360653

[76] Jung J. , Nam J. H. , Park H. W. , Oh U. , Yoon J. H. , and Lee M. G. , Dynamic Modulation Of ANO1/TMEM16A HCO3(-) Permeability By Ca2+/Calmodulin, Proceedings of the National Academy of Sciences of the United States of America. (2013) 110, no. 1, 360–365, 10.1073/pnas.1211594110, 23248295. 23248295 PMC3538232

[77] Song C. , Chai Z. , Chen S. , Zhang H. , Zhang X. , and Zhou Y. , Intestinal Mucus Components And Secretion Mechanisms: What We Do And Do Not Know, Experimental & Molecular Medicine. (2023) 55, no. 4, 681–691, 10.1038/s12276-023-00960-y, 37009791. 37009791 PMC10167328

[78] Stenmark H. , Rab GTPases as Coordinators of Vesicle Traffic, Nature Reviews Molecular Cell Biology. (2009) 10, no. 8, 513–525, 10.1038/nrm2728. 19603039

[79] Chavrier P. , Parton R. G. , Hauri H. P. , Simons K. , and Zerial M. , Localization of Low Molecular Weight GTP Binding Proteins to Exocytic and Endocytic Compartments, Cell. (1990) 62, no. 2, 317–329, 10.1016/0092-8674(90)90369-p, 2115402. 2115402

[80] Ceresa B. P. and Bahr S. J. , rab 7 Activity Affects Epidermal Growth Factor: Epidermal Growth Factor Receptor Degradation by Regulating Endocytic Trafficking From the Late Endosome, Journal of Biological Chemistry. (2006) 281, no. 2, 1099–1106, 10.1074/jbc.M504175200, 16282324. 16282324

[81] Gaur P. , Rajendran Y. , Srivastava B. , Markandey M. , Fishbain-Yoskovitz V. , Mohapatra G. , Suhail A. , Chaudhary S. , Tyagi S. , Yadav S. C. , Pandey A. K. , Merbl Y. , Bajaj A. , Ahuja V. , and Srikanth C. , Rab7-dependent Regulation of Goblet Cell Protein CLCA1 Modulates Gastrointestinal Homeostasis, Elife. (2024) 12, 10.7554/eLife.89776, 38593125. PMC11003743 38593125

[82] Mukherjee S. and Hooper L. V. , Antimicrobial Defense of the Intestine, Immunity. (2015) 42, no. 1, 28–39, 10.1016/j.immuni.2014.12.028. 25607457

[83] Singh R. , Balasubramanian I. , Zhang L. , and Gao N. , Metaplastic Paneth Cells In Extra-Intestinal Mucosal Niche Indicate A Link To Microbiome And Inflammation, Frontiers in Physiology. (2020) 11, 10.3389/fphys.2020.00280, 32296343. PMC7138011 32296343

[84] Ganz T. , Defensins: Antimicrobial Peptides of Innate Immunity, Nature Reviews Immunology. (2003) 3, no. 9, 710–720, 10.1038/nri1180. 12949495

[85] Tang Y. Q. , Yuan J. , Osapay G. , Osapay K. , Tran D. , Miller C. J. , Ouellette A. J. , and Selsted M. E. , A Cyclic Antimicrobial Peptide Produced in Primate Leukocytes by the Ligation of Two Truncated Alpha-Defensins, Science. (1999) 286, no. 5439, 498–502, 10.1126/science.286.5439.498, 10521339. 10521339

[86] Bevins C. L. and Salzman N. H. , Paneth Cells, Antimicrobial Peptides And Maintenance Of Intestinal Homeostasis, Nature Reviews Microbiology. (2011) 9, no. 5, 356–368, 10.1038/nrmicro2546, 21423246. 21423246

[87] Ehmann D. , Wendler J. , Koeninger L. , Larsen I. S. , Klag T. , Berger J. , Marette A. , Schaller M. , Stange E. F. , Malek N. P. , Jensen B. A. H. , and Wehkamp J. , Paneth cell *α*-defensins HD-5 and HD-6 Display Differential Degradation Into Active Antimicrobial Fragments, Proceedings of the National Academy of Sciences. (2019) 116, no. 9, 3746–3751, 10.1073/pnas.1817376116, 30808760. PMC6397583 30808760

[88] Nakamura K. , Sakuragi N. , and Ayabe T. , A Monoclonal Antibody-Based Sandwich Enzyme-Linked Immunosorbent Assay for Detection of Secreted *α*-defensin, Analytical Biochemistry. (2013) 443, no. 2, 124–131, 10.1016/j.ab.2013.08.021, 23994564. 23994564

[89] Masuda K. , Sakai N. , Nakamura K. , Yoshioka S. , and Ayabe T. , Bactericidal Activity of Mouse *α*-Defensin Cryptdin-4 Predominantly Affects Noncommensal Bacteria, Journal of Innate Immunity. (2011) 3, no. 3, 315–326, 10.1159/000322037, 21099205. 21099205

[90] Parks W. C. , Wilson C. L. , and López-Boado Y. S. , Matrix Metalloproteinases as Modulators of Inflammation and Innate Immunity, Nature Reviews Immunology. (2004) 4, no. 8, 617–629, 10.1038/nri1418, 15286728. 15286728

[91] Wilson C. L. , Ouellette A. J. , Satchell D. P. , Ayabe T. , López-Boado Y. S. , Stratman J. L. , Hultgren S. J. , Matrisian L. M. , and Parks W. C. , Regulation of Intestinal Alpha-Defensin Activation by the Metalloproteinase Matrilysin in Innate Host Defense, Science. (1999) 286, no. 5437, 113–117, 10.1126/science.286.5437.113, 10506557. 10506557

[92] Nawaz N. , Wen S. , Wang F. , Nawaz S. , Raza J. , Iftikhar M. , and Usman M. , Lysozyme and Its Application as Antibacterial Agent in Food Industry, Molecules. (2022) 27, no. 19, 10.3390/molecules27196305, 36234848. PMC9572377 36234848

[93] Silhavy T. J. , Kahne D. , and Walker S. , The Bacterial Cell Envelope, Cold Spring Harbor Perspectives in Biology. (2010) 2, no. 5, a000414, 10.1101/cshperspect.a000414, 20452953. 20452953 PMC2857177

[94] Yu S. , Balasubramanian I. , Laubitz D. , Tong K. , Bandyopadhyay S. , Lin X. , Flores J. , Singh R. , Liu Y. , Macazana C. , Zhao Y. , Béguet-Crespel F. , Patil K. , Midura-Kiela M. T. , Wang D. , Yap G. S. , Ferraris R. P. , Wei Z. , Bonder E. M. , Häggblom M. M. , Zhang L. , Douard V. , Verzi M. P. , Cadwell K. , Kiela P. R. , and Gao N. , Paneth Cell-Derived Lysozyme Defines The Composition Of Mucolytic Microbiota And The Inflammatory Tone Of The Intestine, Immunity. (2020) 53, no. 2, 398–416.e8, 10.1016/j.immuni.2020.07.010, 32814028. 32814028 PMC7461615

[95] Han J. , Balasubramanian I. , Flores J. A. , Bandyopadhyay S. , Yang J. , Liu Y. , Singh R. , Setty P. , Kiela P. , Ferraris R. , and Gao N. , Intestinal Lysozyme Engagement of *Salmonella Typhimurium* Stimulates the Release of Barrier-Impairing Inv E and Lpp 1, Journal of Biological Chemistry. (2024) 300, no. 7, 107424, 10.1016/j.jbc.2024.107424, 38823640. 38823640 PMC11255904

[96] Unno M. , Yonekura H. , Nakagawara K. , Watanabe T. , Miyashita H. , Moriizumi S. , Okamoto H. , Itoh T. , and Teraoka H. , Structure, Chromosomal Localization, And Expression Of Mouse Reg Genes, Reg I And Reg II. A Novel Type Of Reg Gene, Reg II, Exists In The Mouse Genome, Journal of Biological Chemistry. (1993) 268, no. 21, 15974–15982, 10.1016/s0021-9258(18)82347-x, 8340418. 8340418

[97] Narushima Y. , Unno M. , Nakagawara K. , Mori M. , Miyashita H. , Suzuki Y. , Noguchi N. , Takasawa S. , Kumagai T. , Yonekura H. , and Okamoto H. , Structure, Chromosomal Localization and Expression of Mouse Genes Encoding Type III Reg, RegIII*α*, RegIII*β*, RegIII*γ* , Gene. (1997) 185, no. 2, 159–168, 10.1016/s0378-1119(96)00589-6, 9055810. 9055810

[98] Abe M. , Nata K. , Akiyama T. , Shervani N. J. , Kobayashi S. , Tomioka-Kumagai T. , Ito S. , Takasawa S. , and Okamoto H. , Identification of a Novel Reg Family Gene, Reg IIIdelta, and Mapping of All Three Types of Reg Family Gene in a 75 Kilobase Mouse Genomic Region, Gene. (2000) 246, no. 1–2, 111–122, 10.1016/s0378-1119(00)00059-7, 10767532. 10767532

[99] Sasaki N. , Sachs N. , Wiebrands K. , Ellenbroek S. I. , Fumagalli A. , Lyubimova A. , Begthel H. , van den Born M. , van Es J. , Karthaus W. R. , Li V. S. , López-Iglesias C. , Peters P. J. , van Rheenen J. , van Oudenaarden A. , and Clevers H. , Reg4+ Deep Crypt Secretory Cells Function as Epithelial Niche for Lgr5+ Stem Cells in Colon, Proceedings of the National Academy of Sciences. (2016) 113, no. 37, E5399–E5407, 10.1073/pnas.1607327113, 27573849. PMC5027439 27573849

[100] Miki T. and Hardt W. D. , Outer Membrane Permeabilization Is an Essential Step in the Killing of Gram-Negative Bacteria by the Lectin RegIIIbeta, PLoS One. (2013) 8, no. 7, e69901, 10.1371/journal.pone.0069901, 23922847. 23922847 PMC3726741

[101] Vaishnava S. , Yamamoto M. , Severson K. M. , Ruhn K. A. , Yu X. , Koren O. , Ley R. , Wakeland E. K. , and Hooper L. V. , The Antibacterial Lectin RegIIIgamma Promotes the Spatial Segregation of Microbiota and Host in the Intestine, Science. (2011) 334, no. 6053, 255–258, 10.1126/science.1209791, 21998396. 21998396 PMC3321924

[102] Zheng Y. , Valdez P. A. , Danilenko D. M. , Hu Y. , Sa S. M. , Gong Q. , Abbas A. R. , Modrusan Z. , Ghilardi N. , de Sauvage F. J. , and Ouyang W. , Interleukin-22 Mediates Early Host Defense Against Attaching and Effacing Bacterial Pathogens, Nature Medicine. (2008) 14, no. 3, 282–289, 10.1038/nm1720, 18264109. 18264109

[103] Kobayashi K. , Honme Y. , and Sashihara T. , *Lactobacillus Delbrueckii Subsp*. *Bulgaricus* 2038 And *Streptococcus Thermophilus* 1131 Induce The Expression Of The REG3 Family In The Small Intestine Of Mice Via The Stimulation Of Dendritic Cells And Type 3 Innate Lymphoid Cells, Nutrients. (2019) 11, no. 12, 10.3390/nu11122998, 31817820. PMC6950248 31817820

[104] Okumura R. , Kurakawa T. , Nakano T. , Kayama H. , Kinoshita M. , Motooka D. , Gotoh K. , Kimura T. , Kamiyama N. , Kusu T. , Ueda Y. , Wu H. , Iijima H. , Barman S. , Osawa H. , Matsuno H. , Nishimura J. , Ohba Y. , Nakamura S. , Iida T. , Yamamoto M. , Umemoto E. , Sano K. , and Takeda K. , Lypd8 Promotes the Segregation of Flagellated Microbiota and Colonic Epithelia, Nature. (2016) 532, no. 7597, 117–121, 10.1038/nature17406, 27027293. 27027293

[105] Okumura R. , Kodama T. , Hsu C. C. , Sahlgren B. H. , Hamano S. , Kurakawa T. , Iida T. , and Takeda K. , Lypd8 Inhibits Attachment of Pathogenic Bacteria to Colonic Epithelia, Mucosal Immunology. (2020) 13, no. 1, 75–85, 10.1038/s41385-019-0219-4, 31659301. 31659301

[106] Hsu C. C. , Okumura R. , Motooka D. , Sasaki R. , Nakamura S. , Iida T. , and Takeda K. , Alleviation of Colonic Inflammation by Lypd8 in a Mouse Model of Inflammatory Bowel Disease, International Immunology. (2021) 33, no. 7, 35972, 10.1093/intimm/dxab012. 33822948

[107] Sender R. , Fuchs S. , and Milo R. , Revised Estimates for the Number of Human and Bacteria Cells in the Body, PLOS Biology. (2016) 14, no. 8, e1002533, 10.1371/journal.pbio.1002533, 27541692. 27541692 PMC4991899

[108] Eckburg P. B. , Bik E. M. , Bernstein C. N. , Purdom E. , Dethlefsen L. , Sargent M. , Gill S. R. , Nelson K. E. , and Relman D. A. , Diversity of the Human Intestinal Microbial Flora, Science. (2005) 308, no. 5728, 1635–1638, 10.1126/science.1110591, 15831718. 15831718 PMC1395357

[109] O′Toole P. W. and Jeffery I. B. , Gut Microbiota and Aging, Science. (2015) 350, no. 6265, 1214–1215, 10.1126/science.aac8469. 26785481

[110] Yatsunenko T. , Rey F. E. , Manary M. J. , Trehan I. , Dominguez-Bello M. G. , Contreras M. , Magris M. , Hidalgo G. , Baldassano R. N. , Anokhin A. P. , Heath A. C. , Warner B. , Reeder J. , Kuczynski J. , Caporaso J. G. , Lozupone C. A. , Lauber C. , Clemente J. C. , Knights D. , Knight R. , and Gordon J. I. , Human Gut Microbiome Viewed Across Age And Geography, Nature. (2012) 486, no. 7402, 222–227, 10.1038/nature11053, 22699611. 22699611 PMC3376388

[111] Odamaki T. , Kato K. , Sugahara H. , Hashikura N. , Takahashi S. , Xiao J. Z. , Abe F. , and Osawa R. , Age-Related Changes In Gut Microbiota Composition From Newborn To Centenarian: A Cross-Sectional Study, BMC Microbiology. (2016) 16, no. 1, 10.1186/s12866-016-0708-5, 27220822. PMC4879732 27220822

[112] Rowland I. , Gibson G. , Heinken A. , Scott K. , Swann J. , Thiele I. , and Tuohy K. , Gut Microbiota Functions: Metabolism Of Nutrients And Other Food Components, European Journal of Nutrition. (2018) 57, no. 1, 1–24, 10.1007/s00394-017-1445-8, 28393285. PMC5847071 28393285

[113] Kamada N. , Kim Y. G. , Sham H. P. , Vallance B. A. , Puente J. L. , Martens E. C. , and Núñez G. , Regulated Virulence Controls The Ability Of A Pathogen To Compete With The Gut Microbiota, Science. (2012) 336, no. 6086, 1325–1329, 10.1126/science.1222195, 22582016. 22582016 PMC3439148

[114] Pike C. M. and Theriot C. M. , Mechanisms Of Colonization Resistance Against Clostridioides Difficile, Journal of Infectious Diseases. (2021) 223, no. Supplement_3, S194–S200, 10.1093/infdis/jiaa408, 33326565. 33326565 PMC8206795

[115] Ubeda C. , Bucci V. , Caballero S. , Djukovic A. , Toussaint N. C. , Equinda M. , Lipuma L. , Ling L. , Gobourne A. , No D. , Taur Y. , Jenq R. R. , van den Brink M. R. , Xavier J. B. , and Pamer E. G. , Intestinal Microbiota Containing Barnesiella Species Cures Vancomycin-Resistant Enterococcus Faecium Colonization, Infection Immunity. (2013) 81, no. 3, 965–973, 10.1128/IAI.01197-12, 23319552. 23319552 PMC3584866

[116] Kamada N. , Chen G. Y. , Inohara N. , and Núñez G. , Control of Pathogens and Pathobionts by the Gut Microbiota, Nature Immunology. (2013) 14, no. 7, 685–690, 10.1038/ni.2608, 23778796. 23778796 PMC4083503

[117] Bajic D. , Niemann A. , Hillmer A. K. , Mejias-Luque R. , Bluemel S. , Docampo M. , Funk M. C. , Tonin E. , Boutros M. , Schnabl B. , Busch D. H. , Miki T. , Schmid R. M. , van den Brink M. R. M. , Gerhard M. , and Stein-Thoeringer C. K. , Gut Microbiota-Derived Propionate Regulates the Expression of Reg3 Mucosal Lectins and Ameliorates Experimental Colitis in Mice, Journal of Crohn′s and Colitis. (2020) 14, no. 10, 1462–1472, 10.1093/ecco-jcc/jjaa065, 32227170. PMC8921751 32227170

[118] Pérez-Reytor D. , Puebla C. , Karahanian E. , and García K. , Use of Short-Chain Fatty Acids for the Recovery of the Intestinal Epithelial Barrier Affected by Bacterial Toxins, Frontiers in Physiology. (2021) 12, 10.3389/fphys.2021.650313, 34108884. PMC8181404 34108884

[119] Li X. , Yao Z. , Qian J. , Li H. , and Li H. , Lactate Protects Intestinal Epithelial Barrier Function From Dextran Sulfate Sodium-Induced Damage by GPR81 Signaling, Nutrients. (2024) 16, no. 5, 10.3390/nu16050582, 38474712. PMC10934655 38474712

[120] Fukuda S. , Toh H. , Hase K. , Oshima K. , Nakanishi Y. , Yoshimura K. , Tobe T. , Clarke J. M. , Topping D. L. , Suzuki T. , Taylor T. D. , Itoh K. , Kikuchi J. , Morita H. , Hattori M. , and Ohno H. , Bifidobacteria Can Protect From Enteropathogenic Infection Through Production of Acetate, Nature. (2011) 469, no. 7331, 543–547, 10.1038/nature09646, 21270894. 21270894

[121] Jacobson A. , Lam L. , Rajendram M. , Tamburini F. , Honeycutt J. , Pham T. , van Treuren W. , Pruss K. , Stabler S. R. , Lugo K. , Bouley D. M. , Vilches-Moure J. G. , Smith M. , Sonnenburg J. L. , Bhatt A. S. , Huang K. C. , and Monack D. , A Gut Commensal-Produced Metabolite Mediates Colonization Resistance to Salmonella Infection, Cell Host & Microbe. (2018) 24, no. 2, 296–307.e7, 10.1016/j.chom.2018.07.002, 30057174. 30057174 PMC6223613

[122] Rivera-Chávez F. , Zhang L. F. , Faber F. , Lopez C. A. , Byndloss M. X. , Olsan E. E. , Xu G. , Velazquez E. M. , Lebrilla C. B. , Winter S. E. , and Bäumler A. J. , Depletion of Butyrate-Producing Clostridia from the Gut Microbiota Drives an Aerobic Luminal Expansion of Salmonella, Cell Host & Microbe. (2016) 19, no. 4, 443–454, 10.1016/j.chom.2016.03.004, 27078066. 27078066 PMC4832419

[123] Zhao Y. , Chen F. , Wu W. , Sun M. , Bilotta A. J. , Yao S. , Xiao Y. , Huang X. , Eaves-Pyles T. D. , Golovko G. , Fofanov Y. , D′Souza W. , Zhao Q. , Liu Z. , and Cong Y. , GPR43 Mediates Microbiota Metabolite SCFA Regulation Of Antimicrobial Peptide Expression In Intestinal Epithelial Cells Via Activation Of Mtor And STAT3, Mucosal Immunology. (2018) 11, no. 3, 752–762, 10.1038/mi.2017.118, 29411774. 29411774 PMC5976519

[124] Gantois I. , Ducatelle R. , Pasmans F. , Haesebrouck F. , Hautefort I. , Thompson A. , Hinton J. C. , and van Immerseel F. , Butyrate Specifically Down-Regulates Salmonella Pathogenicity Island 1 Gene Expression, Applied and Environmental Microbiology Journal. (2006) 72, no. 1, 946–949, 10.1128/AEM.72.1.946-949.2006, 16391141. PMC1352287 16391141

[125] Yu J. , Liu T. , Guo Q. , Wang Z. , Chen Y. , and Dong Y. , Disruption of the Intestinal Mucosal Barrier Induced by High Fructose and Restraint Stress Is Regulated by the Intestinal Microbiota and Microbiota Metabolites, Microbiology Spectrum. (2023) 11, no. 2, e0469822, 10.1128/spectrum.04698-22, 36719201. 36719201 PMC10100858

[126] Shin N. R. , Lee J. C. , Lee H. Y. , Kim M. S. , Whon T. W. , Lee M. S. , and Bae J. W. , An Increase in the *Akkermansia* spp. Population Induced by Metformin Treatment Improves Glucose Homeostasis in Diet-Induced Obese Mice, Gut. (2014) 63, no. 5, 727–735, 10.1136/gutjnl-2012-303839, 23804561. 23804561

[127] Hapfelmeier S. , Lawson M. A. , Slack E. , Kirundi J. K. , Stoel M. , Heikenwalder M. , Cahenzli J. , Velykoredko Y. , Balmer M. L. , Endt K. , Geuking M. B. , Curtiss R. , McCoy K. D. , and Macpherson A. J. , Reversible Microbial Colonization Of Germ-Free Mice Reveals The Dynamics Of Iga Immune Responses, Science. (2010) 328, no. 5986, 1705–1709, 10.1126/science.1188454, 20576892. 20576892 PMC3923373

[128] Takeuchi T. and Ohno H. , Reciprocal Regulation of IgA and the Gut Microbiota: A Key Mutualism in the Intestine, International Immunology. (2021) 33, no. 12, 781–786, 10.1093/intimm/dxab049, 34346497. 34346497

[129] Tezuka H. and Ohteki T. , Regulation of IgA Production by Intestinal Dendritic Cells and Related Cells, Frontiers in Immunology. (2019) 10, 10.3389/fimmu.2019.01891, 31456802. PMC6700333 31456802

[130] Pabst O. and Slack E. , IgA and the Intestinal Microbiota: The Importance of Being Specific, Mucosal Immunology. (2020) 13, no. 1, 12–21, 10.1038/s41385-019-0227-4, 31740744. 31740744 PMC6914667

[131] Huus K. E. , Petersen C. , and Finlay B. B. , Diversity and Dynamism of IgA-Microbiota Interactions, Nature Reviews Immunology. (2021) 21, no. 8, 514–525, 10.1038/s41577-021-00506-1, 33568782. 33568782

[132] Nagaishi T. , Watabe T. , Kotake K. , Kumazawa T. , Aida T. , Tanaka K. , Ono R. , Ishino F. , Usami T. , Miura T. , Hirakata S. , Kawasaki H. , Tsugawa N. , Yamada D. , Hirayama K. , Yoshikawa S. , Karasuyama H. , Okamoto R. , Watanabe M. , Blumberg R. S. , and Adachi T. , Immunoglobulin A-Specific Deficiency Induces Spontaneous Inflammation Specifically In The Ileum, Gut. (2022) 71, no. 3, 487–496, 10.1136/gutjnl-2020-322873, 33963042. 33963042 PMC8809603

[133] Shimada S. , Kawaguchi-Miyashita M. , Kushiro A. , Sato T. , Nanno M. , Sako T. , Matsuoka Y. , Sudo K. , Tagawa Y. I. , Iwakura Y. , and Ohwaki M. , Generation of Polymeric Immunoglobulin Receptor-Deficient Mouse With Marked Reduction of Secretory IgA, Journal of Immunology. (1999) 163, no. 10, 5367–5373, 10.4049/jimmunol.163.10.5367, 10553061. 10553061

[134] Wijburg O. L. , Uren T. K. , Simpfendorfer K. , Johansen F. E. , Brandtzaeg P. , and Strugnell R. A. , Innate Secretory Antibodies Protect Against Natural Salmonella Typhimurium Infection, Journal of Experimental Medicine. (2006) 203, no. 1, 21–26, 10.1084/jem.20052093, 16390940. 16390940 PMC2118088

[135] Ivanov I. I. , Atarashi K. , Manel N. , Brodie E. L. , Shima T. , Karaoz U. , Wei D. , Goldfarb K. C. , Santee C. A. , Lynch S. V. , Tanoue T. , Imaoka A. , Itoh K. , Takeda K. , Umesaki Y. , Honda K. , and Littman D. R. , Induction Of Intestinal Th17 Cells By Segmented Filamentous Bacteria, Cell. (2009) 139, no. 3, 485–498, 10.1016/j.cell.2009.09.033, 19836068. 19836068 PMC2796826

[136] Tanoue T. , Umesaki Y. , and Honda K. , Immune Responses To Gut Microbiota-Commensals And Pathogens, Gut Microbes. (2010) 1, no. 4, 224–233, 10.4161/gmic.1.4.12613, 21327029. 21327029 PMC3023604

[137] Brabec T. , Vobořil M. , Schierová D. , Valter E. , Šplíchalová I. , Dobeš J. , Březina J. , Dobešová M. , Aidarova A. , Jakubec M. , Manning J. , Blumberg R. , Waisman A. , Kolář M. , Kubovčiak J. , Šrůtková D. , Hudcovic T. , Schwarzer M. , Froňková E. , Pinkasová T. , Jabandžiev P. , and Filipp D. , IL-17-Driven Induction of Paneth Cell Antimicrobial Functions Protects the Host From Microbiota Dysbiosis and Inflammation in the Ileum, Mucosal Immunology. (2023) 16, no. 4, 373–385, 10.1016/j.mucimm.2023.01.005, 36739089. 36739089

[138] Lee J. S. , Tato C. M. , Joyce-Shaikh B. , Gulen M. F. , Cayatte C. , Chen Y. , Blumenschein W. M. , Judo M. , Ayanoglu G. , McClanahan T. K. , Li X. , and Cua D. J. , Interleukin-23-Independent IL-17 Production Regulates Intestinal Epithelial Permeability, Immunity. (2015) 43, no. 4, 727–738, 10.1016/j.immuni.2015.09.003, 26431948. 26431948 PMC6044435

[139] Lin X. , Gaudino S. J. , Jang K. K. , Bahadur T. , Singh A. , Banerjee A. , Beaupre M. , Chu T. , Wong H. T. , Kim C. K. , Kempen C. , Axelrad J. , Huang H. , Khalid S. , Shah V. , Eskiocak O. , Parks O. B. , Berisha A. , McAleer J. P. , Good M. , Hoshino M. , Blumberg R. , Bialkowska A. B. , Gaffen S. L. , Kolls J. K. , Yang V. W. , Beyaz S. , Cadwell K. , and Kumar P. , IL-17RA-Signaling In Lgr5+ Intestinal Stem Cells Induces Expression Of Transcription Factor ATOH1 To Promote Secretory Cell Lineage Commitment, Immunity. (2022) 55, no. 2, 237–253.e8, 10.1016/j.immuni.2021.12.016, 35081371. 35081371 PMC8895883

[140] Lee C. , Song J. H. , Cha Y. E. , Chang D. K. , Kim Y. H. , and Hong S. N. , Intestinal Epithelial Responses to IL-17 in Adult Stem Cell-Derived Human Intestinal Organoids, Journal of Crohn′s and Colitis. (2022) 16, no. 12, 1911–1923, 10.1093/ecco-jcc/jjac101, 35927216. 35927216

[141] Wang S. , Gong J. , Wang J. , Wang W. L. , and Huang L. H. , Interleukin-22: The Hub Bridging Gut Homeostasis and Metabolism, Trends in Immunology. (2026) 47, no. 2, 147–159, 10.1016/j.it.2025.10.009, 41266201. 41266201

[142] Keir M. , Yi Y. , Lu T. , and Ghilardi N. , The Role of IL-22 in Intestinal Health and Disease, Journal of Experimental Medicine. (2020) 217, no. 3, e20192195, 10.1084/jem.20192195, 32997932. 32997932 PMC7062536

[143] Pickert G. , Neufert C. , Leppkes M. , Zheng Y. , Wittkopf N. , Warntjen M. , Lehr H. A. , Hirth S. , Weigmann B. , Wirtz S. , Ouyang W. , Neurath M. F. , and Becker C. , STAT3 Links IL-22 Signaling In Intestinal Epithelial Cells To Mucosal Wound Healing, Journal of Experimental Medicine. (2009) 206, no. 7, 1465–1472, 10.1084/jem.20082683, 19564350. 19564350 PMC2715097

[144] Singh A. , Beaupre M. , Villegas-Novoa C. , Shiomitsu K. , Gaudino S. J. , Tawch S. , Damle R. , Kempen C. , Choudhury B. , McAleer J. P. , Sheridan B. S. , Denoya P. , Blumberg R. S. , Hearing P. , Allbritton N. L. , and Kumar P. , IL-22 Promotes Mucin-Type O-Glycosylation And MATH1+ Cell-Mediated Amelioration Of Intestinal Inflammation, Cell Reports. (2024) 43, no. 5, 114206, 10.1016/j.celrep.2024.114206, 38733584. 38733584 PMC11328608

[145] Johansson M. E. , Gustafsson J. K. , Holmén-Larsson J. , Jabbar K. S. , Xia L. , Xu H. , Ghishan F. K. , Carvalho F. A. , Gewirtz A. T. , Sjövall H. , and Hansson G. C. , Bacteria Penetrate The Normally Impenetrable Inner Colon Mucus Layer In Both Murine Colitis Models And Patients With Ulcerative Colitis, Gut. (2014) 63, no. 2, 281–291, 10.1136/gutjnl-2012-303207, 23426893. 23426893 PMC3740207

[146] Jakobsson H. E. , Rodríguez-Piñeiro A. M. , Schütte A. , Ermund A. , Boysen P. , Bemark M. , Sommer F. , Bäckhed F. , Hansson G. C. , and Johansson M. E. , The Composition Of The Gut Microbiota Shapes The Colon Mucus Barrier, EMBO Reports. (2015) 16, no. 2, 164–177, 10.15252/embr.201439263, 25525071. 25525071 PMC4328744

[147] Mann E. R. , Lam Y. K. , and Uhlig H. H. , Short-Chain Fatty Acids: Linking Diet, the Microbiome and Immunity, Nature Reviews Immunology. (2024) 24, no. 8, 577–595, 10.1038/s41577-024-01014-8. 38565643

[148] Reikvam D. H. , Derrien M. , Islam R. , Erofeev A. , Grcic V. , Sandvik A. , Gaustad P. , Meza-Zepeda L. A. , Jahnsen F. L. , Smidt H. , and Johansen F. E. , Epithelial-Microbial Crosstalk in Polymeric Ig Receptor Deficient Mice, European Journal of Immunology. (2012) 42, no. 11, 2959–2970, 10.1002/eji.201242543, 22865203. 22865203

[149] Kumar P. , Monin L. , Castillo P. , Elsegeiny W. , Horne W. , Eddens T. , Vikram A. , Good M. , Schoenborn A. A. , Bibby K. , Montelaro R. C. , Metzger D. W. , Gulati A. S. , and Kolls J. K. , Intestinal Interleukin-17 Receptor Signaling Mediates Reciprocal Control of the Gut Microbiota and Autoimmune Inflammation, Immunity. (2016) 44, no. 3, 659–671, 10.1016/j.immuni.2016.02.007, 26982366. 26982366 PMC4794750

[150] Chassaing B. , Koren O. , Goodrich J. K. , Poole A. C. , Srinivasan S. , Ley R. E. , and Gewirtz A. T. , Dietary Emulsifiers Impact The Mouse Gut Microbiota Promoting Colitis And Metabolic Syndrome, Nature. (2015) 519, no. 7541, 92–96, 10.1038/nature14232, 25731162. 25731162 PMC4910713

[151] Chancharoenthana W. , Kamolratanakul S. , Udompornpitak K. , Wannigama D. L. , Schultz M. J. , and Leelahavanichkul A. , Alcohol-Induced Gut Permeability Defect Through Dysbiosis And Enterocytic Mitochondrial Interference Causing Pro-Inflammatory Macrophages In A Dose Dependent Manner, Scientific Reports. (2025) 15, no. 1, 14710, 10.1038/s41598-025-97593-0, 40289168. 40289168 PMC12034794

[152] Vanuytsel T. , van Wanrooy S. , Vanheel H. , Vanormelingen C. , Verschueren S. , Houben E. , Salim Rasoel S. , Tόth J. , Holvoet L. , Farré R. , van Oudenhove L. , Boeckxstaens G. , Verbeke K. , and Tack J. , Psychological Stress and Corticotropin-Releasing Hormone Increase Intestinal Permeability in Humans by a Mast Cell-Dependent Mechanism, Gut. (2014) 63, no. 8, 1293–1299, 10.1136/gutjnl-2013-305690, 24153250. 24153250

[153] Bjarnason I. , Scarpignato C. , Holmgren E. , Olszewski M. , Rainsford K. D. , and Lanas A. , Mechanisms of Damage to the Gastrointestinal Tract From Nonsteroidal Anti-Inflammatory Drugs, Gastroenterology.(2018) 154, no. 3, 500–514, 10.1053/j.gastro.2017.10.049, 29221664. 29221664

[154] Di Vincenzo F. , Del Gaudio A. , Petito V. , Lopetuso L. R. , and Scaldaferri F. , Gut Microbiota, Intestinal Permeability, and Systemic Inflammation: A Narrative Review, Internal and Emergency Medicine. (2024) 19, no. 2, 275–293, 10.1007/s11739-023-03374-w, 37505311. 37505311 PMC10954893

[155] Ahmad R. , Sorrell M. F. , Batra S. K. , Dhawan P. , and Singh A. B. , Gut Permeability And Mucosal Inflammation: Bad, Good Or Context Dependent, Mucosal Immunology. (2017) 10, no. 2, 307–317, 10.1038/mi.2016.128, 28120842. 28120842 PMC6171348

[156] Kwon J. , Lee C. , Heo S. , Kim B. , and Hyun C. K. , DSS-Induced Colitis Is Associated With Adipose Tissue Dysfunction And Disrupted Hepatic Lipid Metabolism Leading To Hepatosteatosis And Dyslipidemia In Mice, Scientific Reports. (2021) 11, no. 1, 10.1038/s41598-021-84761-1, 33674694. PMC7935975 33674694

[157] Liu H. , Chen R. , Wen S. , Li Q. , Lai X. , Zhang Z. , Sun L. , Sun S. , and Cao F. , Tea (*Camellia sinensis*) Ameliorates DSS-Induced Colitis and Liver Injury by Inhibiting TLR4/NF-*κ*B/NLRP3 Inflammasome in Mice, Biomedicine & Pharmacotherapy. (2023) 158, 114136, 10.1016/j.biopha.2022.114136, 36535201. 36535201

[158] Chen S. N. , Tan Y. , Xiao X. C. , Li Q. , Wu Q. , Peng Y. Y. , Ren J. , and Dong M. L. , Deletion of TLR4 Attenuates Lipopolysaccharide-Induced Acute Liver Injury by Inhibiting Inflammation and Apoptosis, Acta Pharmacologica Sinica. (2021) 42, no. 10, 1610–1619, 10.1038/s41401-020-00597-x, 33495514. 33495514 PMC8463538

[159] Sakai J. , Cammarota E. , Wright J. A. , Cicuta P. , Gottschalk R. A. , Li N. , Fraser I. D. C. , and Bryant C. E. , Lipopolysaccharide-Induced NF-*κ*B Nuclear Translocation Is Primarily Dependent on MyD88, but TNF*α* Expression Requires TRIF and MyD88, Scientific Reports. (2017) 7, no. 1, 10.1038/s41598-017-01600-y, 28469251. PMC5431130 28469251

[160] Takeda K. and Akira S. , TLR Signaling Pathways, Seminars in Immunology. (2004) 16, no. 1, 3–9, 10.1016/j.smim.2003.10.003. 14751757

[161] Zhuang Y. P. , Zhang Y. T. , Zhang R. X. , Zhong H. J. , and He X. X. , The Gut-Liver Axis in Nonalcoholic Fatty Liver Disease: Association of Intestinal Permeability with Disease Severity and Treatment Outcomes, International Journal of Clinical Practice. (2022) 2022, no. 1, 4797453, 10.1155/2022/4797453, 35685554. 35685554 PMC9159210

[162] Soppert J. , Brandt E. F. , Heussen N. M. , Barzakova E. , Blank L. M. , Kuepfer L. , Hornef M. W. , Trebicka J. , Jankowski J. , Berres M. L. , and Noels H. , Blood Endotoxin Levels as Biomarker of Nonalcoholic Fatty Liver Disease: A Systematic Review and Meta-Analysis, Clinical Gastroenterology and Hepatology. (2023) 21, no. 11, 2746–2758, 10.1016/j.cgh.2022.11.030, 36470528. 36470528

[163] Zeng C. , Liu X. , Zhu S. , Xiong D. , Zhu L. , Hou X. , Zou K. , and Bai T. , Resolvin D1 Ameliorates Hepatic Steatosis by Remodeling the Gut Microbiota and Restoring the Intestinal Barrier Integrity in DSS-Induced Chronic Colitis, International Immunopharmacology. (2022) 103, 108500, 10.1016/j.intimp.2021.108500, 34974401. 34974401

[164] Yang A. M. , Inamine T. , Hochrath K. , Chen P. , Wang L. , Llorente C. , Bluemel S. , Hartmann P. , Xu J. , Koyama Y. , Kisseleva T. , Torralba M. G. , Moncera K. , Beeri K. , Chen C. S. , Freese K. , Hellerbrand C. , Lee S. M. L. , Hoffman H. M. , Mehal W. Z. , Garcia-Tsao G. , Mutlu E. A. , Keshavarzian A. , Brown G. D. , Ho S. B. , Bataller R. , Stärkel P. , Fouts D. E. , and Schnabl B. , Intestinal Fungi Contribute to Development of Alcoholic Liver Disease, Journal of Clinical Investigation. (2017) 127, no. 7, 2829–2841, 10.1172/JCI90562, 28530644. 28530644 PMC5490775

[165] Lee H. S. , Kim J. M. , Lee H. L. , Go M. J. , Lee D. Y. , Kim C. W. , Kim H. J. , and Heo H. J. , Eucommia Ulmoides Leaves Alleviate Cognitive Dysfunction In Dextran Sulfate Sodium (DSS)-Induced Colitis Mice Through Regulating JNK/TLR4 Signaling Pathway, International Journal of Molecular Sciences. (2024) 25, no. 7, 10.3390/ijms25074063, 38612870. PMC11012925 38612870

[166] Wasiak J. and Gawlik-Kotelnicka O. , Intestinal Permeability and Its Significance in Psychiatric Disorders - A Narrative Review and Future Perspectives, Behavioural Brain Research. (2023) 448, 114459, 10.1016/j.bbr.2023.114459, 37121278. 37121278

[167] Carloni S. and Rescigno M. , The Gut-Brain Vascular Axis in Neuroinflammation, Seminars in Immunology. (2023) 69, 10.1016/j.smim.2023.101802, 101802. 37422929

[168] Kelly J. R. , Kennedy P. J. , Cryan J. F. , Dinan T. G. , Clarke G. , and Hyland N. P. , Breaking Down the Barriers: The Gut Microbiome, Intestinal Permeability and Stress-Related Psychiatric Disorders, Frontiers in Cellular Neuroscience. (2015) 9, 10.3389/fncel.2015.00392, 26528128. PMC4604320 26528128

[169] Che J. , Sun Y. , Deng Y. , and Zhang J. , Blood-Brain Barrier Disruption: A Culprit Of Cognitive Decline?, Fluids and Barriers of the CNS. (2024) 21, no. 1, 10.1186/s12987-024-00563-3, 39113115. PMC11305076 39113115

[170] Jackson A. , Engen P. A. , Forsyth C. B. , Shaikh M. , Naqib A. , Wilber S. , Frausto D. M. , Raeisi S. , Green S. J. , Bradaric B. D. , Persons A. L. , Voigt R. M. , and Keshavarzian A. , Intestinal Barrier Dysfunction in the Absence of Systemic Inflammation Fails to Exacerbate Motor Dysfunction and Brain Pathology in a Mouse Model of Parkinson′s Disease, Frontiers in Neurology. (2022) 13, 882628, 10.3389/fneur.2022.882628, 35665034. 35665034 PMC9159909

[171] Zonis S. , Pechnick R. N. , Ljubimov V. A. , Mahgerefteh M. , Wawrowsky K. , Michelsen K. S. , and Chesnokova V. , Chronic Intestinal Inflammation Alters Hippocampal Neurogenesis, Journal of Neuroinflammation. (2015) 12, no. 1, 10.1186/s12974-015-0281-0, 25889852. PMC4403851 25889852

[172] Boles J. S. , Krueger M. E. , Jernigan J. E. , Cole C. L. , Neighbarger N. K. , Uriarte Huarte O. , and Tansey M. G. , A Leaky Gut Dysregulates Gene Networks in the Brain Associated With Immune Activation, Oxidative Stress, and Myelination in a Mouse Model of Colitis, Brain, Behavior, and Immunity. (2024) 117, 473–492, 10.1016/j.bbi.2024.02.007, 38341052. 38341052

[173] Lahiri S. , Kim H. , Garcia-Perez I. , Reza M. M. , Martin K. A. , Kundu P. , Cox L. M. , Selkrig J. , Posma J. M. , Zhang H. , Padmanabhan P. , Moret C. , Gulyás B. , Blaser M. J. , Auwerx J. , Holmes E. , Nicholson J. , Wahli W. , and Pettersson S. , The Gut Microbiota Influences Skeletal Muscle Mass and Function in Mice, Science Translational Medicine. (2019) 11, no. 502, 10.1126/scitranslmed.aan5662, eaan5662, 31341063. 31341063 PMC7501733

[174] Saul D. and Kosinsky R. L. , Dextran Sodium Sulfate-Induced Colitis as a Model for Sarcopenia in Mice, Inflammatory Bowel Disease. (2020) 26, no. 1, 56–65, 10.1093/ibd/izz127, 31228348. 31228348

[175] Rashidah N. H. , Lim S. M. , Neoh C. F. , Majeed A. B. A. , Tan M. P. , Khor H. M. , Tan A. H. , Rehiman S. H. , and Ramasamy K. , Differential Gut Microbiota And Intestinal Permeability Between Frail And Healthy Older Adults: A Systematic Review, Ageing Research Reviews. (2022) 82, 10.1016/j.arr.2022.101744, 101744, 36202312. 36202312

[176] Li C. , Li Y. , Wang N. , Ge Z. , Shi Z. , Wang J. , Ding B. , Bi Y. , Wang Y. , and Hong Z. , Intestinal Permeability Associated with the Loss of Skeletal Muscle Strength in Middle-Aged and Older Adults in Rural Area of Beijing, China, Healthcare. (2022) 10, no. 6, 10.3390/healthcare10061100, 35742149. PMC9223217 35742149

[177] Niu Y. , Wang F. , Liu C. , Liu Z. , and Hu X. , Intestinal Amino Acid Metabolic Response And Its Roles In Inflammatory Bowel Disease, Cell Communication And Signaling. (2026) 24, no. 1, 10.1186/s12964-025-02622-0, 41466281. PMC12853750 41466281

[178] White P. J. , McGarrah R. W. , Herman M. A. , Bain J. R. , Shah S. H. , and Newgard C. B. , Insulin Action, Type 2 Diabetes, And Branched-Chain Amino Acids: A Two-Way Street, Molecular Metabolism. (2021) 52, 101261, 10.1016/j.molmet.2021.101261, 34044180. 34044180 PMC8513145

[179] Zhao T. , Fan J. , Abu-Zaid A. , Burley S. K. , and Zheng X. F. S. , Nuclear mTOR Signaling Orchestrates Transcriptional Programs Underlying Cellular Growth and Metabolism, Cells. (2024) 13, no. 9, 10.3390/cells13090781, 38727317. PMC11083943 38727317

[180] Fan J. , Zhang X. , Zhang J. , Zhao T. , Burley S. K. , and Zheng X. F. S. , PDX1 Phosphorylation at S61 by mTORC1 Links Nutrient Signaling to *β* Cell Function and Metabolic Disease, Cell Reports. (2026) 45, no. 1, 116811, 10.1016/j.celrep.2025.116811, 41528843. 41528843 PMC12949489

[181] Fan J. , Yuan Z. , Burley S. K. , Libutti S. K. , and Zheng X. F. S. , Amino Acids Control Blood Glucose Levels Through mTor Signaling, European Journal of Cell Biology. (2022) 101, no. 3, 151240, 10.1016/j.ejcb.2022.151240, 35623230. 35623230 PMC10035058

[182] Fan J. , Khanzada Z. , and Xu Y. , Mechanisms Underlying Muscle-Related Diseases and Aging: Insights into Pathophysiology and Therapeutic Strategies, Muscles. (2025) 4, no. 3, 10.3390/muscles4030026, 40843913. PMC12371960 40843913

[183] Aoki T. , Oyanagi E. , Miyake S. , Hamada H. , Kawashima M. , Watanabe C. , and Yano H. , Anti-inflammatory Properties of Skeletal Muscle Protects Against Muscle Wasting in Colitis of Dextran Sulfate Sodium Model Mice, Kawasaki Journal of Medical Welfare. (2023) 29, no. 1, 23–30.

[184] Mancin L. , Wu G. D. , and Paoli A. , Gut Microbiota-Bile Acid-Skeletal Muscle Axis, Trends in Microbiology. (2023) 31, no. 3, 254–269, 10.1016/j.tim.2022.10.003, 36319506. 36319506

[185] Ranganathan P. , Jayakumar C. , Santhakumar M. , and Ramesh G. , Netrin-1 Regulates Colon-Kidney Cross Talk Through Suppression of IL-6 Function in a Mouse Model of DSS-Colitis, American Journal of Physiology-Renal Physiology. (2013) 304, no. 9, F1187–F1197, 10.1152/ajprenal.00702.2012, 23445618. 23445618 PMC3651630

[186] Rosner M. H. , Reis T. , Husain-Syed F. , Vanholder R. , Hutchison C. , Stenvinkel P. , Blankestijn P. J. , Cozzolino M. , Juillard L. , Kashani K. , Kaushik M. , Kawanishi H. , Massy Z. , Sirich T. L. , Zuo L. , and Ronco C. , Classification of Uremic Toxins and Their Role in Kidney Failure, Clinical Journal of the American Society of Nephrology. (2021) 16, no. 12, 1918–1928, 10.2215/CJN.02660221, 34233920. 34233920 PMC8729494

[187] Vanholder R. , Schepers E. , Pletinck A. , Nagler E. V. , and Glorieux G. , The Uremic Toxicity Of Indoxyl Sulfate And P-Cresyl Sulfate: A Systematic Review, Journal of the American Society of Nephrology. (2014) 25, no. 9, 1897–1907, 10.1681/ASN.2013101062, 24812165. 24812165 PMC4147984

[188] Vaziri N. D. , Yuan J. , and Norris K. , Role Of Urea In Intestinal Barrier Dysfunction And Disruption Of Epithelial Tight Junction In Chronic Kidney Disease, American Journal of Nephrology. (2013) 37, no. 1, 1–6, 10.1159/000345969, 23258127. 23258127 PMC3686571

[189] Wu I. W. , Hsu K. H. , Lee C. C. , Sun C. Y. , Hsu H. J. , Tsai C. J. , Tzen C. Y. , Wang Y. C. , Lin C. Y. , and Wu M. S. , p-Cresyl Sulphate And Indoxyl Sulphate Predict Progression Of Chronic Kidney Disease, Nephrology Dialysis Transplantation. (2011) 26, no. 3, 938–947, 10.1093/ndt/gfq580, 20884620. PMC3042976 20884620

[190] Leong S. C. and Sirich T. L. , Indoxyl Sulfate-Review of Toxicity and Therapeutic Strategies, Toxins. (2016) 8, no. 12, 10.3390/toxins8120358, 27916890. PMC5198552 27916890

[191] Salmenkari H. , Adeshara K. , Pirttiniemi A. , Lindén J. , Lehtonen S. , Sandholm N. , Groop P. H. , and Lehto M. , Dextran Sodium Sulfate-Induced Colitis In Male BALB/C Mice Leads To Albuminuria And Increased Markers Of Inflammation And Tissue Damage In The Kidney, Physiological Reports. (2025) 13, no. 5, e70161, 10.14814/phy2.70161, 40018982. 40018982 PMC11868992

[192] Lau W. L. and Vaziri N. D. , The Leaky Gut and Altered Microbiome in Chronic Kidney Disease, Journal of Renal Nutrition. (2017) 27, no. 6, 458–461, 10.1053/j.jrn.2017.02.010, 29056165. 29056165

[193] Yang Q. , Su S. , Luo N. , and Cao G. , Adenine-Induced Animal Model Of Chronic Kidney Disease: Current Applications And Future Perspectives, Renal Failure. (2024) 46, no. 1, 2336128, 10.1080/0886022X.2024.2336128, 38575340. 38575340 PMC10997364

[194] Hayeeawaema F. , Muangnil P. , Jiangsakul J. , Tipbunjong C. , Huipao N. , and Khuituan P. , A Novel Model Of Adenine-Induced Chronic Kidney Disease-Associated Gastrointestinal Dysfunction In Mice: The Gut-Kidney Axis, Saudi Journal of Biological Sciences. (2023) 30, no. 6, 103660, 10.1016/j.sjbs.2023.103660, 37213695. 37213695 PMC10193294

[195] Macura B. , Kiecka A. , and Szczepanik M. , Intestinal Permeability Disturbances: Causes, Diseases And Therapy, Clinical and Experimental Medicine. (2024) 24, no. 1, 10.1007/s10238-024-01496-9, 39340718. PMC11438725 39340718

[196] Rusticeanu M. , Zimmer V. , and Lammert F. , Visualising and Quantifying Intestinal Permeability -Where Do We Stand, Annals of Hepatology. (2021) 23, 10.1016/j.aohep.2020.09.010, 100266, 33045414. 33045414

[197] Ioannou M. , Borkent J. , Severance E. G. , Yolken R. H. , Fasano A. , Sommer I. E. C. , and Haarman B. C. M. , Biomarkers of Intestinal Permeability in Major Psychiatric Disorders: Distinct Biological Roles Call for a More Nuanced Application, Progress in Neuro-Psychopharmacology & Biological Psychiatry. (2025) 139, 10.1016/j.pnpbp.2025.111405, 111405, 40436362. 40436362

[198] Remund B. , Yilmaz B. , and Sokollik C. , D-Lactate: Implications for Gastrointestinal Diseases, Children. (2023) 10, no. 6, 10.3390/children10060945, 37371177. PMC10297508 37371177

[199] Murray J. , Kok K. B. , and Ayling R. M. , Fecal Calprotectin in Gastrointestinal Disease, Clinical Chemistry. (2023) 69, no. 7, 699–710, 10.1093/clinchem/hvad051. 37228058

[200] Massier L. , Chakaroun R. , Kovacs P. , and Heiker J. T. , Blurring The Picture In Leaky Gut Research: How Shortcomings Of Zonulin As A Biomarker Mislead The Field Of Intestinal Permeability, Gut. (2021) 70, no. 9, 1801–1802, 10.1136/gutjnl-2020-323026, 33037053. 33037053 PMC8355880

[201] Yang C. and Merlin D. , Unveiling Colitis: A Journey through the Dextran Sodium Sulfate-induced Model, Inflammatory Bowel Disease. (2024) 30, no. 5, 844–853, 10.1093/ibd/izad312, 38280217. PMC11063560 38280217

[202] Ikeda E. , Yamaguchi M. , and Kawabata S. , Gut Microbiota-mediated Alleviation of Dextran Sulfate Sodium-induced Colitis in Mice, Gastro Hep Advances. (2024) 3, no. 4, 461–470, 10.1016/j.gastha.2024.01.016, 39131720. 39131720 PMC11308119

[203] Wang X. , Du C. , Subramanian S. , Turner L. , Geng H. , Bu H. F. , and Tan X. D. , Severe Gut Mucosal Injury Induces Profound Systemic Inflammation And Spleen-Associated Lymphoid Organ Response, Frontiers in Immunology. (2024) 14, 1340442, 10.3389/fimmu.2023.1340442, 38259439. 38259439 PMC10800855

[204] Manzanares L. D. , Dehghani Z. , Herrnreiter C. J. , Ren X. , Sumagin M. E. , Serdiukova A. , Naqib A. , Green S. , Wechsler J. B. , and Sumagin R. , Repeated Dextran Sulfate Sodium Exposure Elicits Distinct Immune Responses Reflecting Human Ulcerative Colitis, Cellular and Molecular Gastroenterology and Hepatology. (2026) 20, no. 4, 101714, 10.1016/j.jcmgh.2025.101714, 41453640. 41453640 PMC12892045

[205] Trefts E. , Gannon M. , and Wasserman D. H. , The Liver, Current Biology. (2017) 27, no. 21, R1147–R1151, 10.1016/j.cub.2017.09.019, 29112863. 29112863 PMC5897118

[206] Volta U. , Bonazzi C. , Bianchi F. B. , Baldoni A. M. , Zoli M. , and Pisi E. , IgA Antibodies to Dietary Antigens in Liver Cirrhosis, Research In Clinic And Laboratory. (1987) 17, no. 3, 235–242, 10.1007/BF02912537, 3671996. 3671996

[207] Solga S. F. and Diehl A. M. , Gut Flora-Based Therapy in Liver Disease? The Liver Cares About the Gut, Hepatology. (2004) 39, no. 5, 1197–1200, 10.1002/hep.20220, 15122746. 15122746

[208] Garcia-Mateo S. , Rondinella D. , Ponziani F. R. , Miele L. , Gasbarrini A. , Cammarota G. , Lanas Á. , and Gomollón F. , Gut Microbiome and Metabolic Dysfunction-Associated Steatotic Liver Disease: Pathogenic Role and Potential for Therapeutics, Best Practice & Research Clinical Gastroenterology. (2024) 72, 101924, 10.1016/j.bpg.2024.101924, 39645278. 39645278

[209] Huang D. Q. , Wong V. W. S. , Rinella M. E. , Boursier J. , Lazarus J. V. , Yki-Järvinen H. , and Loomba R. , Metabolic Dysfunction-Associated Steatotic Liver Disease in Adults, Nature Reviews Disease Primers. (2025) 11, no. 1, 10.1038/s41572-025-00599-1. 40050362

[210] Loomba R. , Friedman S. L. , and Shulman G. I. , Mechanisms And Disease Consequences Of Nonalcoholic Fatty Liver Disease, Cell. (2021) 184, no. 10, 2537–2564, 10.1016/j.cell.2021.04.015, 33989548. 33989548 PMC12168897

[211] Zhu X. , Cai J. , Wang Y. , Liu X. , Chen X. , Wang H. , Wu Z. , Bao W. , Fan H. , and Wu S. , A High-Fat Diet Increases the Characteristics of Gut Microbial Composition and the Intestinal Damage Associated with Non-Alcoholic Fatty Liver Disease, International Journal of Molecular Sciences. (2023) 24, no. 23, 16733, 10.3390/ijms242316733, 38069055. 38069055 PMC10706137

[212] Alsegiani A. S. and Shah Z. A. , The Influence Of Gut Microbiota Alteration On Age-Related Neuroinflammation And Cognitive Decline, Neural Regeneration Research. (2022) 17, no. 11, 2407–2412, 10.4103/1673-5374.335837, 35535879. 35535879 PMC9120705

[213] Fukudo S. , Nomura T. , Muranaka M. , and Taguchi F. , Brain-Gut Response to Stress and Cholinergic Stimulation in Irritable Bowel Syndrome. A Preliminary Study, Journal of Clinical Gastroenterology. (1993) 17, no. 2, 133–141, 10.1097/00004836-199309000-00009, 8031340. 8031340

[214] Cryan J. F. , O′Riordan K. J. , Cowan C. S. M. , Sandhu K. V. , Bastiaanssen T. F. S. , Boehme M. , Codagnone M. G. , Cussotto S. , Fulling C. , Golubeva A. V. , Guzzetta K. E. , Jaggar M. , Long-Smith C. M. , Lyte J. M. , Martin J. A. , Molinero-Perez A. , Moloney G. , Morelli E. , Morillas E. , O′Connor R. , Cruz-Pereira J. S. , Peterson V. L. , Rea K. , Ritz N. L. , Sherwin E. , Spichak S. , Teichman E. M. , van de Wouw M. , Ventura-Silva A. P. , Wallace-Fitzsimons S. E. , Hyland N. , Clarke G. , and Dinan T. G. , The Microbiota-Gut-Brain Axis, Physiological Reviews. (2019) 99, no. 4, 1877–2013, 10.1152/physrev.00018.2018. 31460832

[215] Chen G. , Shi F. , Yin W. , Guo Y. , Liu A. , Shuai J. , and Sun J. , Gut Microbiota Dysbiosis: The Potential Mechanisms By Which Alcohol Disrupts Gut And Brain Functions, Frontiers in Microbiology. (2022) 13, 916765, 10.3389/fmicb.2022.916765, 35966709. 35966709 PMC9372561

[216] Scisciola L. , Fontanella R. A. , Surina C. V. , Paolisso G. , and Barbieri M. , Sarcopenia and Cognitive Function: Role of Myokines in Muscle Brain Cross-Talk, Life. (2021) 11, no. 2, 10.3390/life11020173, 33672427. PMC7926334 33672427

[217] Fried L. P. , Tangen C. M. , Walston J. , Newman A. B. , Hirsch C. , Gottdiener J. , Seeman T. , Tracy R. , Kop W. J. , Burke G. , and McBurnie M. A. , Frailty in Older Adults: Evidence for a Phenotype, Journals of Gerontology, Series A: Biological Sciences and Medical Sciences. (2001) 56, no. 3, M146–M157, 10.1093/gerona/56.3.m146. 11253156

[218] Xue Q. L. , Bandeen-Roche K. , Varadhan R. , Zhou J. , and Fried L. P. , Initial Manifestations of Frailty Criteria and the Development of Frailty Phenotype in the Women′s Health and Aging Study II, Journals of Gerontology, Series A: Biological Sciences and Medical Sciences. (2008) 63, no. 9, 984–990, 10.1093/gerona/63.9.984, 18840805. 18840805

[219] Bodine S. C. , Stitt T. N. , Gonzalez M. , Kline W. O. , Stover G. L. , Bauerlein R. , Zlotchenko E. , Scrimgeour A. , Lawrence J. C. , Glass D. J. , and Yancopoulos G. D. , Akt/mTOR Pathway Is a Crucial Regulator of Skeletal Muscle Hypertrophy and Can Prevent Muscle Atrophy In Vivo, Nature Cell Biology. (2001) 3, no. 11, 1014–1019, 10.1038/ncb1101-1014, 11715023. 11715023

[220] Loffing J. , Verrey F. , and Wagner C. A. , The Kidneys Matter, Pflugers Archiv European Journal of Physiology. (2022) 474, no. 8, 755–757, 10.1007/s00424-022-02737-0, 35895104. 35895104 PMC9338890

[221] GBD Chronic Kidney Disease Collaboration , Global, Regional, And National Burden Of Chronic Kidney Disease, 1990-2017: A Systematic Analysis For The Global Burden Of Disease Study 2017, Lancet. (2020) 395, no. 10225, 709–733, 10.1016/S0140-6736(20)30045-3, 32061315. 32061315 PMC7049905

[222] Okumura R. and Takeda K. , The Role Of The Mucosal Barrier System In Maintaining Gut Symbiosis To Prevent Intestinal Inflammation, Seminars in Immunopathology. (2024) 47, no. 1, 10.1007/s00281-024-01026-5, 39589551. PMC11599372 39589551

[223] Nascimento D. S. M. D. , Mota A. C. C. C. , Carvalho M. C. D. C. , Andrade E. D. O. , Oliveira É. P. S. F. , Galvão L. L. P. , and Maciel B. L. L. , Can Diet Alter the Intestinal Barrier Permeability in Healthy People? A Systematic Review, Nutrients. (2024) 16, no. 12, 10.3390/nu16121871, 38931225. PMC11206284 38931225

[224] Wellens J. , Vanderstappen J. , Hoekx S. , Vissers E. , Luppens M. , Van Elst L. , Lenfant M. , Raes J. , Derrien M. , Verstockt B. , Ferrante M. , Verbeke K. , Matthys C. , Vermeire S. , and Sabino J. , Effect of Five Dietary Emulsifiers on Inflammation, Permeability, and the Gut Microbiome: A Placebo-Controlled Randomized Trial, Clinical Gastroenterology and Hepatology. (2026) 24, no. 4, 1092–1101, 10.1016/j.cgh.2025.08.005, 40816342. 40816342

[225] Kreimeyer H. , Llorente C. , and Schnabl B. , Influence of Alcohol on the Intestinal Immune System, Alcohol Research. (2025) 45, no. 1, 10.35946/arcr.v45.1.03, 40151622. PMC11913448 40151622

[226] Ghorbani Z. , Shoaibinobarian N. , Noormohammadi M. , Taylor K. , Kazemi A. , Bonyad A. , Khoshdooz S. , Löber U. , and Forslund-Startceva S. K. , Reinforcing Gut Integrity: A Systematic Review and Meta-Analysis of Clinical Trials Assessing Probiotics, Synbiotics, and Prebiotics on Intestinal Permeability Markers, Pharmacological Research. (2025) 216, 10.1016/j.phrs.2025.107780, 107780, 40378939. 40378939

[227] Liu X. , Zhao H. , and Wong A. , Accounting For The Health Risk Of Probiotics, Heliyon. (2024) 10, no. 6, e27908, 10.1016/j.heliyon.2024.e27908, 38510031. 38510031 PMC10950733

[228] Mukhopadhya I. and Louis P. , Gut Microbiota-Derived Short-Chain Fatty Acids And Their Role In Human Health And Disease, Nature Reviews Microbiology. (2025) 23, no. 10, 635–651, 10.1038/s41579-025-01183-w, 40360779. 40360779

[229] Hansen J. K. , Israelsen M. , Nishijima S. , Stinson S. E. , Andersen P. , Johansen S. , Hansen C. D. , Brol M. J. , Klein S. , Schierwagen R. , Uschner F. E. , Sulek K. , Villesen I. F. , Lindvig K. P. , Thorhauge K. H. , Torp N. , Jensen J. M. , Keller M. I. , Jensen G. H. , Detlefsen S. , Leeming D. J. , Stankevic E. , Suvitaival T. , Zawadzki A. , Kuhn M. , Jensen L. J. , Karsdal M. , Trebicka J. , Israelsen H. , Legido-Quigley C. , Bork P. , Arumugam M. , Hansen T. , Thiele M. , and Krag A. , The Postbiotic ReFerm® Versus Standard Nutritional Support in Advanced Alcohol-Related Liver Disease (GALA-POSTBIO): A Randomized Controlled Phase 2 Trial, Nature Communications. (2025) 16, no. 1, 10.1038/s41467-025-60755-9, 40595534. PMC12214853 40595534

[230] Song G. , Xie Y. , Yi L. , Cheng W. , Jia H. , Shi W. , Liu Q. , Fang L. , Xue S. , Liu D. , Zhu J. , and Zhao S. , Bile Acids Affect Intestinal Barrier Function Through FXR and TGR5, Frontiers in Medicine. (2025) 12, 1607899, 10.3389/fmed.2025.1607899, 40692955. 40692955 PMC12277261

[231] Hou L. , Wang H. , Yan M. , Cai Y. , Zheng R. , Ma Y. , Tang W. , and Jiang W. , Obeticholic Acid Attenuates the Intestinal Barrier Disruption in a Rat Model of Short Bowel Syndrome, Biochimica et Biophysica Acta - Molecular Basis of Disease. (2024) 1870, no. 5, 10.1016/j.bbadis.2024.167221, 167221, 38718845. 38718845

[232] Neurath M. F. , Strategies for Targeting Cytokines in Inflammatory Bowel Disease, Nature Reviews Immunology. (2024) 24, no. 8, 559–576, 10.1038/s41577-024-01008-6. 38486124

[233] Cicala M. , Gori M. , Balestrieri P. , Altomare A. , Tullio A. , Di Cola S. , Dejongh S. , Graziani M. G. , Pagnini C. , Carotti S. , Perrone G. , Ribolsi M. , Fiorani M. , Guarino M. P. L. , and Farré R. , Colonic Epithelial Permeability to Ions Is Restored After Vedolizumab Treatment and May Predict Clinical Response in Inflammatory Bowel Disease Patients, International Journal of Molecular Sciences. (2024) 25, no. 11, 10.3390/ijms25115817, 38892004. PMC11172326 38892004

[234] La Torre D. , Van Oudenhove L. , Vanuytsel T. , and Verbeke K. , Psychosocial Stress-Induced Intestinal Permeability In Healthy Humans: What Is The Evidence?, Neurobiology of Stress. (2023) 27, 100579, 10.1016/j.ynstr.2023.100579, 37842017. 37842017 PMC10569989

[235] Li G. , Gao M. , Zhang S. , Dai T. , Wang F. , Geng J. , Rao J. , Qin X. , Qian J. , Zuo L. , Zhou M. , Liu L. , and Zhou H. , Sleep Deprivation Impairs Intestinal Mucosal Barrier by Activating Endoplasmic Reticulum Stress in Goblet Cells, American Journal of Pathology. (2024) 194, no. 1, 85–100, 10.1016/j.ajpath.2023.10.004, 37918798. 37918798

[236] Karl J. P. , Whitney C. C. , Wilson M. A. , Fagnant H. S. , Radcliffe P. N. , Chakraborty N. , Campbell R. , Hoke A. , Gautam A. , Hammamieh R. , and Smith T. J. , Severe, Short-Term Sleep Restriction Reduces Gut Microbiota Community Richness But Does Not Alter Intestinal Permeability In Healthy Young Men, Scientific Reports. (2023) 13, no. 1, 10.1038/s41598-023-27463-0, 36604516. PMC9816096 36604516

